# The Internal Transcribed Spacer (ITS) Region and *trnhH-psbA* Are Suitable Candidate Loci for DNA Barcoding of Tropical Tree Species of India

**DOI:** 10.1371/journal.pone.0057934

**Published:** 2013-02-27

**Authors:** Abhinandan Mani Tripathi, Antariksh Tyagi, Anoop Kumar, Akanksha Singh, Shivani Singh, Lal Babu Chaudhary, Sribash Roy

**Affiliations:** 1 Genetics and Molecular Biology Division, Council for Scientific and Industrial Research-National Botanical Research Institute, Lucknow, Uttar Pradesh, India; 2 Plant Diversity, Systematic and Herbarium Division, Council for Scientific and Industrial Research-National Botanical Research Institute, Lucknow, Uttar Pradesh, India; Montreal Botanical Garden, Canada

## Abstract

**Background:**

DNA barcoding as a tool for species identification has been successful in animals and other organisms, including certain groups of plants. The exploration of this new tool for species identification, particularly in tree species, is very scanty from biodiversity-rich countries like India. *rbcL* and *matK* are standard barcode loci while ITS, and *trnH-psbA* are considered as supplementary loci for plants.

**Methodology and Principal Findings:**

Plant barcode loci, namely, *rbcL*, *matK*, ITS, *trnH-psbA,* and the recently proposed ITS2, were tested for their efficacy as barcode loci using 300 accessions of tropical tree species. We tested these loci for PCR, sequencing success, and species discrimination ability using three methods. *rbcL* was the best locus as far as PCR and sequencing success rate were concerned, but not for the species discrimination ability of tropical tree species. ITS and *trnH-psbA* were the second best loci in PCR and sequencing success, respectively. The species discrimination ability of ITS ranged from 24.4 percent to 74.3 percent and that of *trnH-psbA* was 25.6 percent to 67.7 percent, depending upon the data set and the method used. *matK* provided the least PCR success, followed by ITS2 (59. 0%). Species resolution by ITS2 and *rbcL* ranged from 9.0 percent to 48.7 percent and 13.2 percent to 43.6 percent, respectively. Further, we observed that the NCBI nucleotide database is poorly represented by the sequences of barcode loci studied here for tree species.

**Conclusion:**

Although a conservative approach of a success rate of 60–70 percent by both ITS and *trnH-psbA* may not be considered as highly successful but would certainly help in large-scale biodiversity inventorization, particularly for tropical tree species, considering the standard success rate of plant DNA barcode program reported so far. The recommended *matK* and *rbcL* primers combination may not work in tropical tree species as barcode markers.

## Introduction

The recently concluded Fourth International Conference on DNA barcode held at Adelaide, Australia emphasized on DNA barcoding of tree species. The main aim of barcoding tree species is to check the illegal trade of timbers as well as CITES-listed tree species. Forest trees are fast disappearing worldwide, especially in the developing countries. This is due to deforestation and urbanization and meeting various human needs. In a recent study, it has been predicted that over half of the estimated 11,000 Amazonian tree species may face a direct risk of extinction [1].Thus, there is an urgent need to carry out an inventory and manage diversity using innovative tools, like DNA barcoding. Significant progress has been made in mapping the Neotropical plants during the last decades [2]–[5], including studies on DNA barcoding in tree species [6] and other plant species [7]–[9]. However, such studies on tree species of India are lacking. Large-scale biodiversity inventories are critically needed in order to develop conservation strategies for the diverse ecosystems of India. It has been suggested that the application of new tools, like DNA barcoding, may help identify species with a high confidence, which would be useful in a wide array of applications, including discriminating forest species and large-scale biodiversity surveys [10]–[14].

The DNA barcoding technique utilizes a short fragment of DNA sequence to identify the species. Since its first report in 2003 [10], it has been successfully established in animals as a tool to identify species and taxonomic clarification [10]. A portion of the mitochondrial cytochrome c oxidase 1 (COI or cox1) gene sequence is currently being used as a universal barcode in certain animal groups [10], [11], [15], [16], fungi [17], diatoms [18], and red algae [19]. However, COI has proved to be an unsuitable barcoding marker in land plants [20]–[22], primarily because of the low nucleotide substitution rates of the plant mitochondrial genome [23]. The plastid DNA sequences have been the focus for DNA barcodes for plants. The success rate using plastid markers has not been so high, compared to animals. This is mainly because there are certain inherent difficulties for some groups of plants, which have been well discussed as far as the barcoding of these groups is concerned [24]–[27]. After evaluating the performance of seven leading candidate plastid DNA regions (*atpF–atpH*, *psbK–psbI*, *trnH–psbA* spacers and *matK*, *rbcL*, *rpoB*, *rpoC1* genes), the Plant Working Group of the CBOL recommended the two-marker combination *rbcL*/*matK* as the standard DNA barcodes for plants [13] and, later on, added ITS and *trnH-psbA* as supplementary barcode loci. Yet, the screening for single or multiple regions from plastid and nucleus, appropriate for DNA barcoding in different plant groups, has been an important research focus around the globe.

The four loci, namely, *matK*, *rbcL*, ITS, and *trnH-psbA* have been studied in great detail in taxonomic and floristic contexts as the barcode loci for plants [6], [13], [25], [27]–[34]. Each region has its own strengths and weaknesses to be considered before establishing the universal barcode loci for plants. While *rbcL* remained as the most effective loci in terms of sequence quality and recovery, but not in species discrimination ability [6], [13], [25], *matK* was found to be effective in species discrimination in some cases [7], [13], [21], [29], [33], but not in others [25], [35], [36]. Moreover, in most cases, sequence recovery and the quality of *matK* was not as effective as that with *rbcL* [7], [25], [37]. Similarly, there are mixed reports for the consideration of *trnH-psbA* as barcode locus, some advocating in favor [14], [25], [36]–[39] and others disregarding it [13], [40]. The nuclear region that has so far been considered as barcode loci is the ITS. Though initially, this locus was not advocated by many due to its inherent difficulties–for example, the existence of paralogs, chances of fungal contamination, difficulties in sequence recovery and so on, as discussed [41]–yet, recently, ITS has been reported to be an efficient barcode locus by many [25], [33], [42], [43]. This is mainly due to the advantages gained in using ITS, which outweigh its limitations. More recently, ITS2–part of the ITS region–has been shown as the best barcode locus due to its higher species discrimination and sequence recovery ability across different plant groups [44]–[46]. Thus, these studies indicate that there is a need to further validate these loci among different plant groups in order to choose the most favorable one or their combination thereof. Though the effectiveness of these candidate barcode loci has been discussed, most of them were concerned with herbaceous or shrubs taxa, as mentioned above, and only a few studies are reported on tree and other long living taxa [36], [42], [47], [48]. As echoed by many, for example, Gonzalez et al. [6], we assume that DNA barcoding in tropical trees may be challenging as compared to temperate plants. Moreover, DNA extraction from tropical trees is sometime difficult due to the greater abundance of secondary metabolites [49]. Further, it has been shown that woody plant lineages show consistently lower rates of molecular evolution as compared to herbaceous plant lineages [50], suggesting that the application of DNA barcoding concept should be more difficult for tree floras than for non-woody floras [7], [27]. Applications of DNA barcoding in the tropical trees, especially from India, are still unexplored.

**Figure 1:**
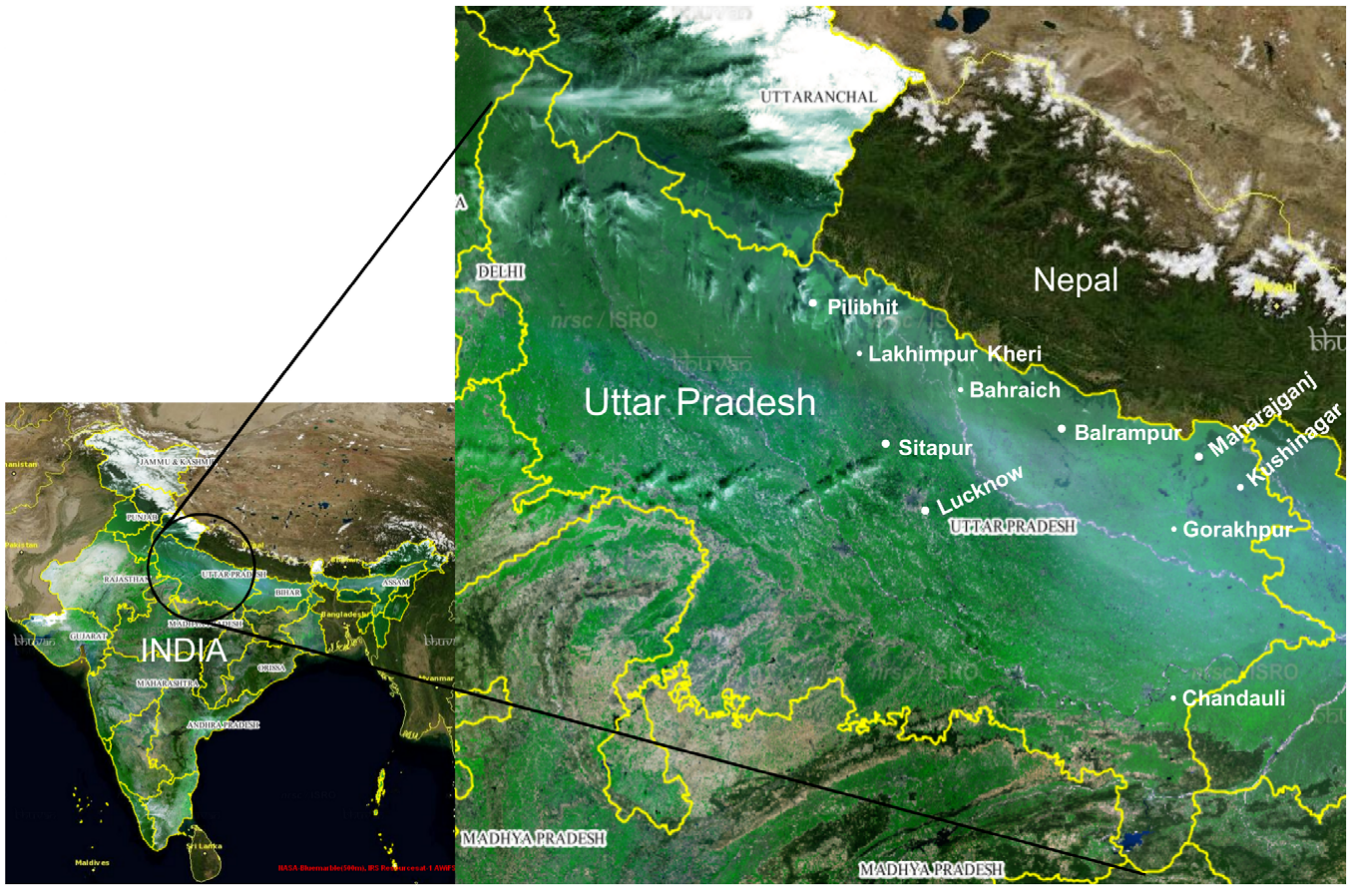
Satellite map of India and province of Uttar Pradesh. Dots indicate places of sample collection. A total of 300 specimens covering 149 species, 82 genera, and 38 families of trees were collected from different places.

India is considered to be one of the 17 mega biodiverse countries of the world, with four biodiversity hotspots. There are around 19,294 species of flowering plants in India of which around 2,560 species are estimated to be tree species [51]. The country has been divided into 12 bio-geographical zones. The samples were collected from the National Botanic Garden (NBG), Lucknow, as well as other parts of the province of Uttar Pradesh (Figure 1). The NBG at Lucknow (Uttar Pradesh) lies at 26° 55′ N, 80°59′ E and at an altitude of 113 m above the mean sea level. The region falls under the range of the bio geographic zone of the Gangetic Plain, which has a typical moist tropical climate. The garden, spread over an area of 65 acres, is a repository of germplasms collected from the various parts of the country as well as some exotic collections. It harbors approximately 5,000 taxa, out of which about 345 species belong to trees. The repository represents almost all the tree species of Uttar Pradesh, which consists of approximately 400 tree species. Initially, we examined 236 accessions using all the five loci for PCR, sequencing, and species discrimination ability. ITS and *trnH-psbA* provided the best species resolution using this data. Then, we added another 64 accessions for ITS and *trnH-psbA* to determine the species discrimination ability in 300 accessions by these loci only. The representative tree species from the NBG as well as from the other parts of the province have been selected to examine the efficacy of standard and proposed barcode loci (ITS, *matK*, *rbcL*, *trnH-psbA,* and ITS2) in tropical tree species.

## Results

### Comparative Performance of Each Barcode Loci

**Figure 2:**
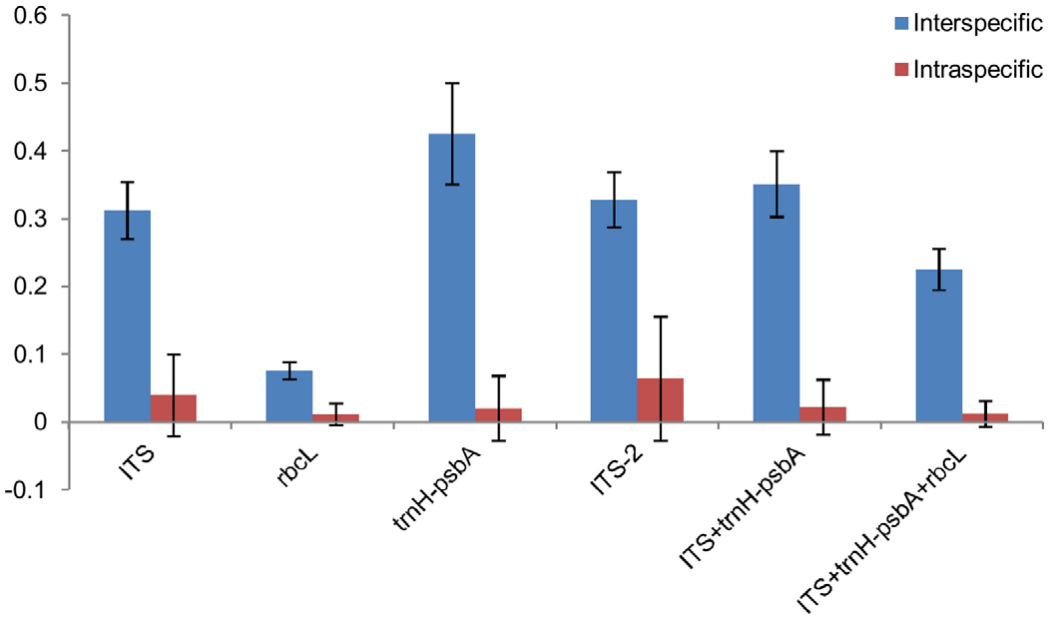
Average inter- and intraspecific genetic divergence of different loci using data set 2. Average inter- and intra-specific genetic divergences were calculated based on p-distance using MEGA5.0 with pairwise deletion options.

**Figure 3:**
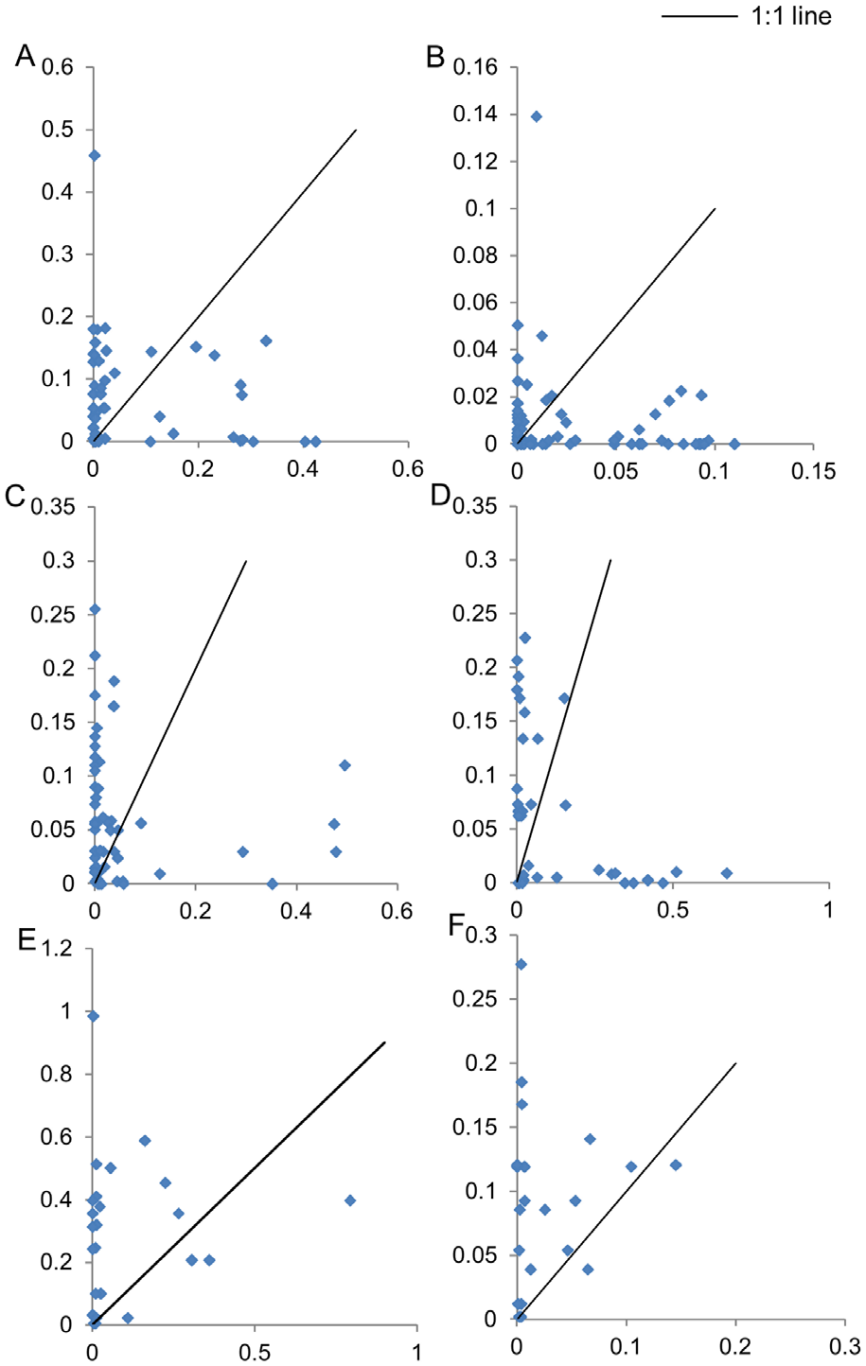
Presence/Absence of barcode gap. Minimum inter-specific and maximum intra-specific p-distances for different loci were calculated for the species having multiple accessions (data set 2).A, ITS; B, rbcL; C, *trnH-psbA*; D, ITS2; E, ITS+*trnH-psbA*, F, ITS+*trnH-psbA+rbcL* X-axis: maximum intra-specific, Y-axis: minimum inter-specific p-distances.

We sampled a total of 300 specimens of tree species. Genomic DNA was extracted from all the accessions. The number of species and sequences analyzed (GenBank accession numbers JX856395-JX856981) under each loci and their comparative performance is depicted in Table 1. Samples were grouped into three sets: set 1, included all the samples; set 2 consists of the species with multiple accessions (irrespective of congeneric species); and set 3 comprised the congener species with multiple accessions in each species. The PCR was performed using all the 300 accessions. However, sequencing was done for 236 accessions using *rbcL, matK,* ITS2 and for 300 accessions using ITS and *trnH-psbA*. Thus, the PCR success percentage was calculated based on 300 accessions for all the loci and the sequencing success rate was calculated based on 236 accessions for *rbcL, matK,* and ITS2 and 300 accessions for ITS and *trnH-psbA*. The PCR and sequencing success rate were highly variable amongst the loci. The *rbcL* showed the highest PCR and sequencing success rate at 87.7 percent and 90.8 percent, respectively. The next best PCR success rate was exhibited by ITS and *trnH-psbA* (74.0%), followed by ITS2 (59.0%), KIM-*matK* (42%), *matK*-NBRI (39%), and *matK* 2.1a (29%). The sequencing success rate of *trnH-psbA* was 78.0 percent, followed by ITS2 (60.0%) and ITS (62.0%). Since the PCR success rate was too low for all the three *matK* primers, these were not evaluated further. The PCR and sequencing percent success was calculated based on three attempts for each failed sample. The highest mean sequence length was provided by *rbcL* (607.6 bp to 618.0 bp, depending on the data set), followed by ITS (582.0 bp to 592.3 bp), *trnH-psbA* (377.9 bp to 407.8 bp), and ITS2 (361.3 bp to 376.1 bp). The percent PIC and percent variable sites for all the loci were variable, depending on the data sets (Table 1). The genetic divergence within and between the species was calculated for data sets 2 and 3 (Figure 2). In data set 2, the average inter-specific divergence was the highest for *trnH-psbA,* followed by ITS2, ITS, and *rbcL*. In data set 3, the average inter-specific divergence was the highest for *trnH-psbA,* followed by ITS, ITS2, and *rbcL*. On the other hand, *rbcL* and *trnH-psbA* showed the lowest intra-specific divergence in data sets 2 and 3, respectively. The genetic divergence of data set 1 samples was not calculated due to the presence of several singleton species. Kruskal-Wallis test between the inter-specific distances of different loci showed *trnH-psbA* as the most divergent locus at the inter-specific level, followed by ITS. *rbcL* was found to be the least divergent at the inter-specific level using both sets 2 and 3 (Table S3 A and C). At the intra-specific level, *rbcL* and *trnH-psbA* were found to be the least divergent loci using data sets 2 and 3 (Table S3 B and D). Wilcoxon matched-pair test between the minimum inter-specific and the maximum intra-specific p-distances of a locus showed that *trnH-psbA* had significantly higher inter-specific distances than intra-specific distances for data set 2 (Table S4 A), but not with data set 3 (Table S4 B). The combination of ITS/*trnH-psbA* also showed significantly higher inter-specific distances than intra-specific distances for data set 2. The intra-specific p-distances were found to be higher than inter-specific p-distances in *rbcL,* using both the data sets (Table S4 A and B). To evaluate the barcoding gap, we looked at the minimum inter- and the maximum intra-specific divergences for each locus. No distinct barcoding gap was noticed in any of the four loci (Figure 3).

**Table 1:** Summary statistics of the standard barcode loci using 300 samples of tree species**.**

Locus	ITS (Set-1)	ITS (Set-2)	ITS (Set-3)	*rbcL (set-1)*	*rbcL* *(set-2)*	*rbcL* *(set-3)*	*trnH-psbA* *(set-1)*	*trnH-psbA* *(set-2)*	*trnH-psbA* *(set-3)*	ITS2(set-1)	ITS2(set-2)	ITS2(Set-3)	KIM-*matK*	*matK*-NBRI	*matK* 2.1a
Number of samples/species attempted	300/148			236/116			300/148			236/116			236/116	236/116	236/116
Number of sequences/species analyzed	153/88	113/48	68/29	210/103	181/74	123/48	189/94	152/60	86/34	100/56	79/35	43/18	27/0		
Number of sequences from GenBank	14	14	6	21	21	14	15	14	8	15	15	6			
Mean sequence length	582.0(107.0)	577.7(115.4)	592.3(109.0)	607.6(61.0)	609.8(56.8)	618.0(39.0)	382.7(106.0)	384.8(103.0)	407.8(107.8)	362.9(81.8)	361.3(83.3)	376.1(76.9)			
% PCR success	74.0			87.7			74.0			59.0			42.0	39.0	29.0
% Sequencing success#	62.0			90.8			78.0			60.0			27.0		
% PIC*	42.7	75.25	66.4	35.4	33.6	29.7	42.27	45.8	41.7	61.8	53.5	53.6			
% Variable sites	54.4	85.8	79.6	45.8	42.8	40.1	57.3	57.0	50.1	69.7	58.0	59.6			

† Figures in parenthesis indicate standard deviation.

# Percent sequencing success refers to the fraction of sequences having QV above 30 and at least 70% overlap between sequence reads using forward and reverse primers (except in a few cases) of total number of PCR products.

* Parsimony informative characters.

### Species Identification

**Figure 4:**
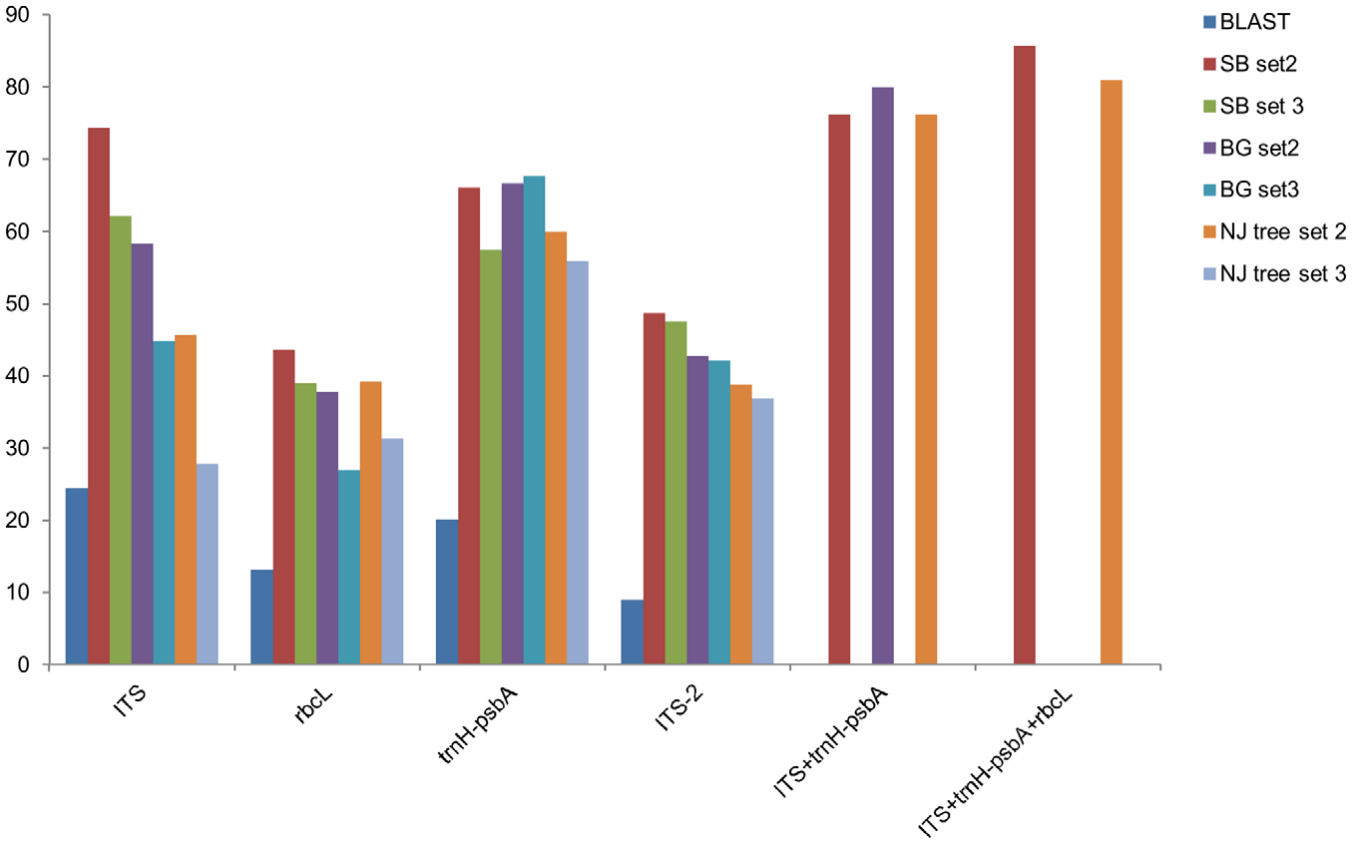
Percent species discrimination ability (Y-axis) of different loci (X-axis) using different methods and data sets. Abbreviations are SB; stand alone blast, BG; barcoding gap, NJ; neighbour joining.

For species identification, we followed three approaches, namely, similarity-based approach (standalone BLAST and BLASTn), barcoding gap, and neighbor-joining (NJ) tree (Figure 4). In the standalone BLAST approach, when data set 2 was used as query sequences against the data set 1 database, the highest species identification was provided by the ITS at 74.3 percent, followed by *trnH-psbA* (66.1%), ITS2 (48.7), and *rbcL* (43.6%). When data set 3 was used as query sequences, ITS showed the highest species resolution of 62.1 percent, followed by *trnH-psbA* (57.4%), *rbcL* (39.0%), and ITS2 (47.6%). We did not use set 1 for standalone BLAST as query sequences against itself because it has several species with single accession. For data set 1, we used BLASTn for species identification. In this method, the correct species assignment was found to be very low by all the tested loci. The highest correct species identification was provided by ITS at 24.4 percent, followed by *trnH-psbA* (20.1%), *rbcL* (13.2%), and ITS2 (9.0). Species resolution efficacy by all the tested loci was further examined using barcoding gap principle for data sets 2 and 3. In this approach, a species is considered to be resolved if the minimum inter-specific divergence is higher than the maximium intra-specific divergence of the species. Following this principle, the highest species resolution was observed for *trnH-psbA* using both data sets 2 and 3 (66.6% and 67.7%, respectively), followed by ITS (58.3% and 44.8%), ITS2 (42.8% and 42.1%), and *rbcL* (37.8%, and 27.0%) (Figure 4). In NJ tree method, a species was considered to be resolved if the accessions under the species form a monophyletic group. In this method, the species discrimination was the highest for *trnH-psbA* using both data sets 2 and 3 (60.0% and 55.9%, respectively) (Figure 4, S1 E and S1 F) while ITS provided the second-highest species resolution using both data sets 2 and 3 (56.2% and 44.8%, respectively) (Figure 4, S1 A and S1 B). The species resolution by ITS2 were 45.7 percent and 27.7 percent, using both data sets 2 and 3, respectively (Figure 4, S1 G and S1 H), and *rbcL* provided 39.1 percent and 31.2 percent species resolution with data set 2 and data set 3, respectively (Figure 4, S1C and D), following the NJ tree method.

**Figure 5:**
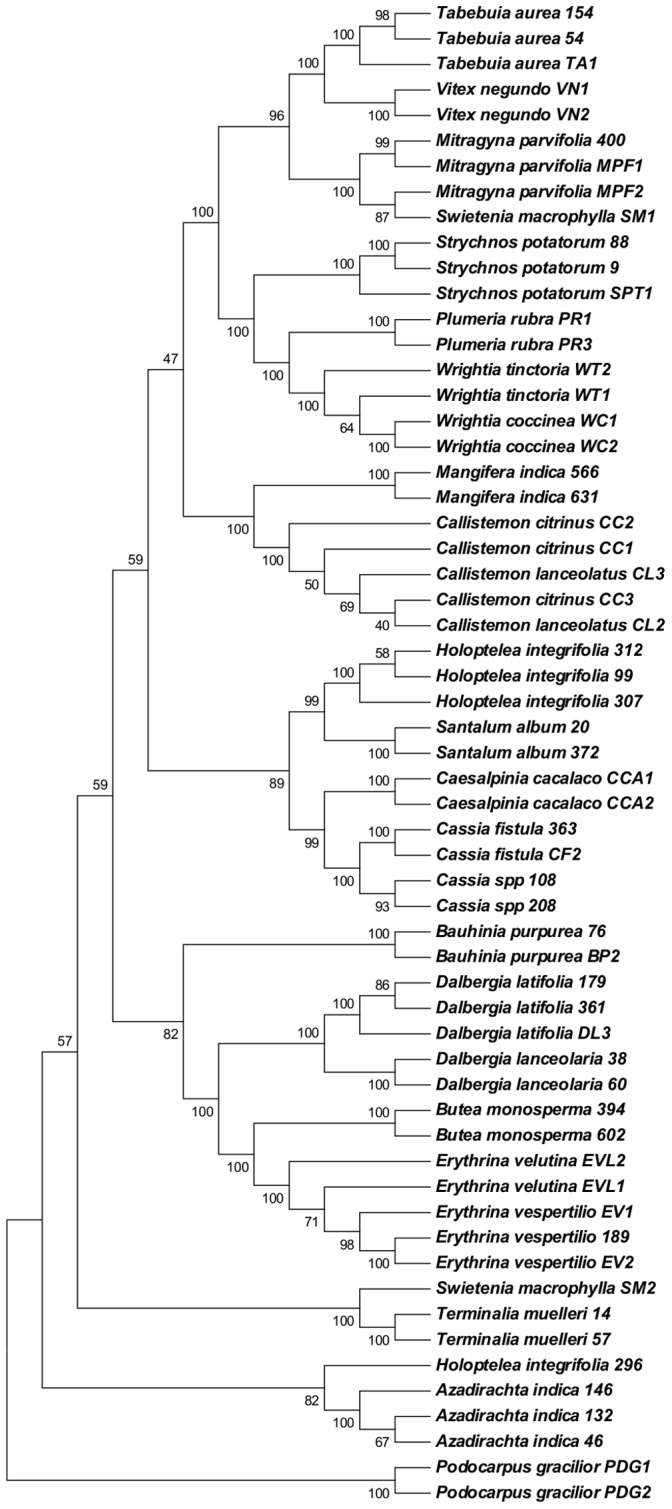
Strict consensus unrooted NJ tree with 1000 bootstrap replicates based on sequence combination of ITS and *trnH-psbA*. Numbers at the branch nodes are bootstrap values. Codes preceding the species name indicate DNA numbers corresponding to the accession numbers analyzed in this study.

All five markers could not be sequenced for exactly the same individuals. Therefore, we could not test the species resolution by various multilocus combinations. However, since ITS and *trnH-psbA* provided the best species resolution and had common accessions for 25 species, with multiple accessions for each species, we examined these two loci combinations using these sequences. Following the barcoding gap approach this combination of loci provided higher species resolution (80.0%) than the individual locus (58.3% for ITS and 66.6% for *trnH-psbA* ) (Figure 4 and 5). Similarly, in the tree-based approach too, this combination provided a higher species resolution (76.2%) (Figure 4 and 5) than the individual locus (56.2% for ITS and 60.0% for *trnH-psbA*). Overall, the combination of ITS/*trnH-psbA* provided the highest species resolution across all the methods. Finally, we evaluated species resolution ability of three loci combination of ITS, *trnH-psbA* and *rbcL* for the species having multiple accessions (21 species, 49 accessions). The three loci combination provided 80.95% and 85.71% species resolution following NJ tree method (Figure S1 I) and barcoding gap principle (Figure 4), respectively.

## Discussion

We examined the efficacy of five barcode loci in identifying the tropical tree species. This is the first report on the evaluation of barcode loci in tropical tree species from India, which is considered one of the mega bio-diverse countries in the world.

An ideal DNA barcode must have adequate conserved regions for universal primer design, high PCR amplification efficiency, and enough variability to be used for species identification [13]. The PCR success rate for all the loci was variable. The striking observation was the extremely low PCR success using *matK* primers. The three different primer pairs of *matK* were not able to provide more than 50 percent PCR success. There are mixed reports about PCR and sequencing success using *matK* primers, depending upon the use of particular primer/s and data sets [25]. This and other studies suggest that existing *matK* primers may not be suitable for plant barcode purposes in general, and tree species, in particular. We are not able to comment on the species identification ability of the *matK* in tree species based on our present study.

The PCR success rate using the *trnH-psbA* was lower than *rbcL* and ITS. This is in contrast to earlier findings, where PCR amplification (as well as species discrimination ability) of *trnH-psbA* was high enough to be considered as barcode loci [7], [8], [13], [21], [22], [37]. The higher rate of PCR amplification success in some of these reports may be attributed to the sampling strategies, which were restricted to particular taxon, or to sampling, which were very shallow in some cases. However, in the CBOL plant working group study involving large numbers of taxa, it was observed that *trnH-psbA* possess attributes that are highly desirable in a plant DNA barcoding system, such as universality, sequence quality and coverage, and discrimination ability [13]. The plant working group rejected this locus because of difficulties in bidirectional sequencing and also due to its higher length (>1000 bp) in some monocots [14] and conifers [52]. We did not increase our effort to obtain a higher PCR amplification rate of *trnH-psbA* using different PCR conditions. But, if efforts are exerted in this direction, like *matK*, the PCR amplification rate may be increased to suit the universality criterion. Finally, the use of the *trnH-psbA* locus has been much criticized because it is prone to errors during sequencing [53]. However, we were able to get high-quality bidirectional sequences with the success rate falling next to *rbcL,* which is considered the most efficient barcode locus as far as sequence quality is considered. The high quality bidirectional sequence was also obtained by Maia et al. in the family Bromeliaceae, where only 2 out of 101 samples failed in generating high quality *trnH-psbA* bidirectional sequences after the second trial [9]. Except for the low PCR amplification rate of *trnH-psbA*, our study agrees with the findings of the work reported by Gonzalez et al. [6], where they observed that *trnH-psbA* was the best performing locus for barcoding Amazonian trees. We emphasize here that, to the best of our knowledge, the study of Gonzalez et al. was the only study involving a large number of tropical tree species for tree barcoding. Our study also indicates that *trnH-psbA* can be considered as a potential tree barcode marker, especially for tropical tree species, with a success rate of species identification ranging from 57.4 percent to 67.7 percent, depending on the method and data set used.

The only nuclear-originated barcode locus that has gained popularity is the internal transcribed spacer region (ITS). The ITS provided the highest species resolution ability among all the five tested loci using tree-based and standalone BLAST approaches with data set 2. The ITS sequences have been proposed as a barcode locus for plants for some time [22]. It was recently suggested as an additional marker by CBOL [13]. ITS has been validated as an efficient barcode locus for identifying species in many groups [31], [42], [44], [54]–[56], including ashes [44] and other tree genera, such as *Cedrela* [42] and *Quercus* [39]. One of the most extensive latest barcoding studies also identified ITS as the best alternative to *matK* and *rbcL* [44]. In our earlier study, ITS provided the best species resolution in the tree *Ficus* and in the shrub *Gossypium* [25]. However, other studies described its inherent difficulties, for example, low PCR success [6], [14], [37], [44], problem of secondary structure formation resulting in poor quality sequence data [57], [58], and multiple copy numbers [59], [60] etc. In our study too, the sequencing success rate was low. In the study by Gonzalez et al. [6], ITS provided a low sequencing success rate (41.0%), but a higher species discrimination rate (80.3%) of tropical Amazonian forest trees. Another major apprehension that has been shown in using ITS as the plant barcode marker is the chance of contamination with endophytic fungus. As reported by Chen et al. [44], we also did not find any fungal sequences when we retrieved sequences after BLAST. In all cases, the best match retrieved the plant species, either as the same plant species sequence as query sequences or as the other plant species. Secondly, we looked for the presence of the characteristic conserved motif in the 5.8S rRNA gene of angiosperm plant ITS sequences [61]. The characteristic motif (5′-GAATTGCAGAAT***C***C-3′) was found in all the ITS sequences whereas the variant of the motif generally found in fungi (5′-GAATTGCAGAAT***T***C-3′) was not found in any of the sequences. Though we did not study the occurrence of paralogous ITS sequences, none of the PCR products showed multiple bands nor were the sequences unreadable, in a fashion easily attributable to the presence of multiple divergent copies. Because these copies generally evolve in a concerted fashion, leading to a single detectable sequence per plant. In order to consider ITS as the barcode locus, these potential problems and their remedies have been further discussed by Hollingsworth [41]. In the present study too, though we did not observe many of the difficulties associated with ITS as a barcode marker, the low sequencing success rate needs to be dealt with.

As an alternative to complete the ITS (ITS1-5.8S-ITS2) region, ITS2 has been suggested as a barcode locus [44], [46]. While one of these suggestions was based on *in silico* analysis [46], another study compared seven candidate DNA barcodes (psbA-trnH, *matK*, *rbcL*, rpoC1, ycf5, ITS2, and ITS) from medicinal plant species and found ITS2 to be the most suitable region (more than 6,600 plant samples belonging to 4,800 species from 753 distinct genera tested), with 92.7 percent discrimination ability at the species level [44]. Following these, other studies also reported about the usefulness of this locus as a barcode marker [31], [62], [63]. We observed a low PCR (59.0%) and sequencing success rate (60.0%) using these primers, whereas species resolution was found to be only 42.8 percent, 48.7 percent, and 9.0 percent using the barcoding gap principle, standalone BLAST, and BLASTn methods, respectively. In evaluating candidate DNA barcoding loci for the economically important timber species of the Mahogany family (Meliaceae), Muellner et al. observed that ITS2 is less variable and had fewer PICs as compared to ITS1. Similarly, Liu et al. did not find ITS2 to be a suitable marker for species identification in *Taxus* lineages, where only 5 of 11 *Taxus* lineages were discriminated by ITS2 [56]. More recently, Sun et al. [64] observed a low PCR success rate by using ITS and ITS2 in *Dioscorea*. Though ITS2 has some other advantages, for example, ease of routine sequencing (as observed in the present study) and conserved length, as compared to ITS1 [44], echoing Hollingsworth et al. [38], we assume that further sampling is needed, involving a wider range of plant species, to assess the potentiality of ITS2 as the barcode loci for land plants.

It is now well established that a universal DNA barcode for plants may be elusive. Therefore, the necessity for a multilocus approach has been suggested [13], [22], [42], [52], [65]. Though we did not try all possible combinations in this study, a two loci combination of ITS and *trnH-psbA* provided better species resolution in the barcode gap method as well as the tree-based approach than when used alone. A number of studies relying on *trnH–psbA* alone [66] or in combination with other regions [48], [67], [68] have confirmed the utility and efficacy of this region for plant barcoding [7]. Hollingsworth et al. [52] recently summarized the seven empirical studies published till date that have involved comparisons of multiple regions in a barcoding context [7], [8], [21], [22], [37], [40], [52]. In most of these studies, ITS was not considered as a component of the multilocus system, whereas *trnH-psbA* was one of the main loci in most cases. In our earlier work too, we found that the combination of these two loci worked better than other combinations in species resolution of the Indian *Berberis* [25]. More recently, Li et al. [33] showed that adding ITS to the multilocus system took the levels of species discrimination success from 50 percent to 62 percent for two or three marker plastid barcodes, to between 77 percent and 82 percent, when ITS was combined with two plastid markers. The higher success rate of species resolution that we observed here using the combination of ITS and *trnH-psbA* (80.0%) may be due to the lower number of species in this data (25 species). Though the three loci combination of ITS, *trnH-psbA* and *rbcL* provided slightly higher species resolution ability (may be due to less number of species than two loci combination) as that of two loci combination of ITS and *trnH-psbA,*for obvious reason of cost effectiveness, the two loci combination is preferred here than three loci combination.

One of the important observations was the very low species identification by all the loci in NCBI BLAST analysis, though other studies have reported higher species resolution in the BLAST approach [36], [44]. This prompted us to check the availability of a specific locus sequence of a particular species in the NCBI database. Our analysis suggests that 68.0 percent and 61.3 percent of the studied species were not represented by the *trnH-psbA* and ITS sequences, respectively, in the NCBI database (Table S5). This could be one of the reasons for the low species identification rate using NCBI BLAST. This further emphasized the need to enrich the NCBI database with nucleotide sequences of the different barcode loci of tree species to be effectively used as the standard barcode reference database.

Overall, although there were differences in species resolution, depending on the methods and data set used for a particular locus, considering the best possible representative data (data set 2, having higher species numbers with more than one accession), popular methods (barcode gap and tree-based), species discrimination ability, and results of our statistical analysis of inter- and intra-specific divergence, *trnH-psbA* and ITS, both can be considered as potential barcode loci for tropical tree species. This is in line with the standard success rate of plant species discrimination level at 60–72 percent, as reported in most of the earlier studies [6], [13], [29], [36].

Tropical forests play a key role in developing large-scale biodiversity inventories and conservation strategies. The DNA barcode of tropical tree species, as reflected here, may not be as difficult as presumed [6]. For example, DNA extraction is expected to be more difficult in tropical plants, due to the greater abundance of secondary metabolites [69], which may affect the overall performance of DNA barcoding. However, we did not find any difficulties in DNA extraction except in case of *Shorea robusta* and *Delbergia sissoo.* These two samples required slight modifications in the extraction protocol as described in materials and methods. Moreover, the high PCR and sequencing success rate by *rbcL* reflect that good quality DNA could be obtained from of tropical tree species for DNA barcoding. Thus, the low PCR and sequencing success rate by *matK* and ITS2 may not pertain to DNA extraction and quality of DNA, rather due to non specificity of primer sequences. Though the species discrimination rate here is not very high yet, the identification of ∼70 percent of tree species by ITS and *trnH-psbA* alone, following the tree-based approach, holds promise as a good candidate marker for tropical tree species identification. This is considering the present success rate of not more than 60–72 percent in a large number of plant species barcode studies reported so far. Further large-scale studies on tropical tree species are needed to establish that the combination of ITS and *trnH-psbA* is better for barcoding tropical trees. This is more important because the combination of a nuclear marker and a plastid marker has an added advantage: the nuclear marker, being inherited from both parents, would provide much more information than an organellar marker alone, which is inherited from only one parent. The *matK* and *rbcL* combination may not work as the ideal barcode loci for tropical tree species, as indicated in the present study and other studies involving tree species [42], [52].

## Materials and Methods

### Sample Collections

We sampled 300 specimens covering 149 species, 82 genera, and 38 families of trees. Specimen vouchers were deposited at the Herbarium of CSIR-National Botanical Research Institute, Lucknow, India (LWG). All necessary permits for the collection of plant specimens were taken from the Principal Chief Conservator of Uttar Pradesh, wherever required. The permission for the specimens of the NBG was granted by the Director, CSIR-NBRI. All collected material was verified by a taxonomic expert. Leaf samples, either fresh or desiccated in silica gel, were used for DNA extraction. The species list, along with accession numbers for each sample, is given in Table S1. Collected samples were grouped into three sets: set 1, which included all the samples; set 2 comprised the species with multiple accessions (irrespective of congeneric species); and set 3 comprised the congener species with multiple accessions in each species. These grouping were done to evaluate the intra-specific divergence using only congener species and also for the application of different methods to estimate the species resolution ability of the different loci more effectively. For example, data set 1 is more suited for BLASTn analysis whereas data sets 2 and 3 are more suited to the application of barcode gap principle and so on. Besides these samples, we also included a few available sequences from the NCBI database for each locus to make up multiple accessions for a species (Table 1).

### DNA Extraction, PCR Amplification, and Sequencing of Candidate DNA Barcodes

Genomic DNA was extracted from either fresh or silica gel-dried leaf materials using Nucleospin plant II Kit (Macherey-Nagel, Germany) according to the manufacturer's instructions and/or the CTAB method with slight modifications in some cases. For example, extraction with choloroform isoamyl alcohol (24∶1) was repeated twice in these cases. We evaluated the most commonly used four barcode loci recommended by CBOL: ITS, *matK*, *rbcL,* and *trnH-psbA*. For *matK,* we tested three primers: KIM-*matK*, *matK*2.1a, 3.2r, and *matK*-NBRI (modified forward primer of *matK*2.1a) (Table S2). PCR amplification was performed in 50-µl reaction mixtures containing approximately 50–75 mg genomic DNA templates, 1.5 mM MgCl_2_, 0.2 mM of each dNTP, 1 µM of each primer, 0.1 mg BSA/ml, and 1 unit *Taq* DNA polymerase. The thermo cycler program was 94°C for 5 minutes (1 cycle), 94°C for 40 seconds, 48°C–52°C (depending upon primer sets used), 72°C for 40 seconds (35 cycles), and 72°C for 5 minutes (1 cycle). The PCR products were cleaned by Qiaquick® PCR Purification kit (Qiagen, Germany). All the loci yielded single amplicon after PCR for almost all the samples. However, if there was more than single amplicon (very few cases), the expected amplicon size was eluted from the gel using the gel extraction kit (Macherey-Nagel, Germany). Cycle sequencing reactions were performed in 10 µl reactions using 1 µl of Big Dye Terminator cycle sequencing chemistry (v3.1; ABI, UK) and run on a automated capillary sequencer, ABI3730XL DNA analyzer (Applied Biosystems, UK). Sequencing was performed in both the directions for each locus.

### Sequence Quality Control and Alignment for Data Analysis

Sequencing was done in both the forward and reverse directions. Base calling was carried out using the Phred program (version no. 0.020425.c). The generated sequences were post processed using software, Gene mapper4.0. Using a 20 bp segments size with 4 bp showing <20 QV were trimmed and the post-trim lengths should be at least 60% of the original read length. A minimum average QV of 30 was considered as the quality sequence. Pairwise alignments were made by using the sequences obtained from the forward and reverse primers. Sequences which covered more than 70 percent overlap between the forward and reverse sequences were considered (except a few sequences where the coverage was less than 50%). DNA sequences were edited manually by the visual inspection of the electropherograms of both the end sequences using Sequencher 4.1.4. Multiple sequence alignment was performed using ClustalW.

### Data Analysis

Several methods have been used for the analysis of barcode data and species resolution. Among others, phylogenetic analysis [27], [56], [66], [70], [71], similarity approaches such as BLAST [37], [72], and approaches based on the barcoding gap principle [13], [73] are the most commonly used for DNA barcode data analysis. We employed these three methods to test the efficacy of barcode loci in the identification of tropical tree species. Similarity-based BLAST is probably the most commonly used method practiced for classifying DNA sequences [74]. It is an algorithm for comparing query sequences with an unaligned reference database calculating pairwise alignments in the process. In similarity-based approaches, we tested two methods, namely, standalone BLAST and BLASTn. Briefly, in standalone BLAST, a self database was prepared consisting of data set 1 and then the query sequences of data sets 2 and 3 were BLAST against it. The ability of a query sequence to identify other sequences of the same species was considered as successful identification. In BLASTn at GenBank, the ID is that of the species associated with the best BLAST hit. This corresponds to choosing the top hit in the BLAST results. In BLAST approaches, we used data set 1 to discriminate between species. In the barcoding gap approach, the minimum inter-specific p-distance and maximum intra-specific p-distance were determined for data sets 2 and 3 using MEGA 5.0. [75] In this approach, a species is considered to be resolved if it’s minimum inter-specific distance is greater than maximum intra-specific distance [13].

The third method was based on the nearest neighbor that relies on NJ trees. We used only NJ tree (Figure 5) because it has been shown that it is a more robust and reliable method with different data sets [76]. Moreover, this method has been reported as fast and accurate both for examining the relationships among species and also to assign unidentified samples to known species [10]. The pairwise p-distance was estimated for each barcode loci and in conjunction with the NJ algorithm of tree reconstruction (NJ tree was constructed using MEGA 5.0 [75]. Bootstrap analyses were based on 1,000 replicates in all cases. To estimate whether a species is resolved, for a given genomic region or combination, we scored how well supported the monophyly of individual species was in bootstrap analysis. We used a cut off of 50% to define support for “successful” resolution as a monophyletic species. We then determined the proportion of well-supported species as a percentage of total species. Other complex methods, such as maximum likelihood, Bayesian and so on, would not result in better taxa discrimination if the intra-specific divergence was equal or higher than the inter-specific divergence or if the inter-specific divergence was nil [10], [77].

The Kruskal-Wallis test with Dunn's multiple comparison was performed to compare the average inter- and intra-specific variability of all the loci. The DNA barcoding gaps were evaluated by comparing the minimum inter-specific divergence and the maximum intra-specific divergence by carrying out the Wilcoxon matched pair test.

## Supporting Information

Figure S1 **Strict consensus unrooted NJ tree based on sequences of different locus and data set used.** Numbers at the branch nodes are bootstrap values. Codes preceding the species name indicate DNA numbers corresponding to the accession numbers analyzed in this study. A) ITS, data set 2; B) ITS data set 3; C) *rbcL*, data set 2; D) *rbcL*, data set 3; E) *trnH-psbA*, data set 2 F) *trnH-psbA*, data set 3; G) ITS2, data set 2; H) ITS2, data set 3; I) ITS+*trnH-psbA+rbcL*.
(PDF)

Table S1 **List of tree species, accession numbers and DNA tube numbers used in this study.**
(XLSX)

Table S2 **List of primer sequences (5′-3′) and their references used in this study.**
(PDF)

Table S3 **Kruskal-Wallis test with Dunn's multiple comparison to compare inter (A) and intraspecific (B) variability for each individual locus.**
(DOCX)

Table S4 **Wilcoxon matched-pair test to compare between minimum inter and maximum intraspecific p-distance differences of different loci. Set 2, (A); Set 3, (B).**
(DOCX)

Table S5 **The status of ITS and** ***trnH-psbA*** **sequences with respect to studied tree species in NCBI nucleotide database as on 03.01.2013.** The table shows the species name whose ITS and/or *trnH-psbA* sequences are abscent in the database.
(DOCX)
