# Correction: The Internal Transcribed Spacer (ITS) Region and *trnhH-psbA* Are Suitable Candidate Loci for DNA Barcoding of Tropical Tree Species of India

**DOI:** 10.1371/journal.pone.0105914

**Published:** 2014-08-11

**Authors:** The PLOS ONE Staff

“*trnhH-psbA*” in the title contains an extra letter. The correct title is: The Internal Transcribed Spacer (ITS) Region and *trnH-psbA* Are Suitable Candidate Loci for DNA Barcoding of Tropical Tree Species of India. The correct citation is: Tripathi AM, Tyagi A, Kumar A, Singh A, Singh S, et al. (2013) The Internal Transcribed Spacer (ITS) Region and *trnH-psbA* Are Suitable Candidate Loci for DNA Barcoding of Tropical Tree Species of India. PLoS ONE 8(2): e57934. doi:10.1371/journal.pone.0057934.
