# Supplementary material for: The Internal Transcribed Spacer (ITS) Region and trnhH-psbA Are Suitable Candidate Loci for DNA Barcoding of Tropical Tree Species of India
Source: PLoS One. 2013 Feb 27;8(2):e57934. doi: 10.1371/journal.pone.0057934 (PMC3584017; doi:10.1371/journal.pone.0057934)
Supplement: Figure S1 — Strict consensus unrooted NJ tree based on sequences of different locus and data set used. Numbers at the branch nodes are bootstrap values. Codes preceding the species name indicate DNA numbers corresponding to the accession numbers analyzed in this study. A) ITS, data set 2; B) ITS data set 3; C) rbcL, data set 2; D) rbcL, data set 3; E) trnH-psbA, data set 2 F) trnH-psbA, data set 3; G) ITS2, data set 2; H) ITS2, data set 3; I) ITS+trnH-psbA+rbcL. (PDF) [file pone.0057934.s001.pdf]

A. ITS set 2

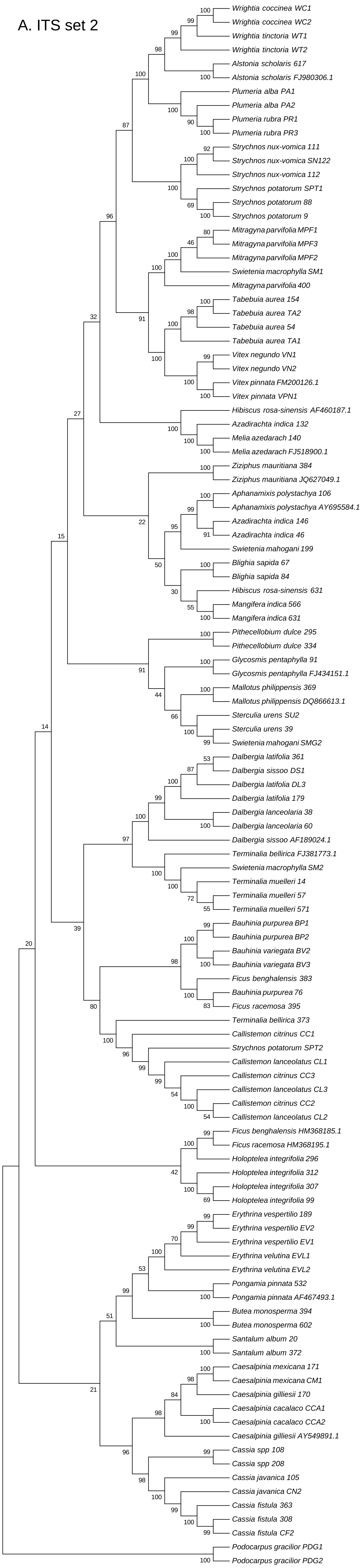
Wrightia coccinea WC1
Wrightia coccinea WC2
Wrightia tinctoria WT1
Wrightia tinctoria WT2
Alstonia scholaris 617
Alstonia scholaris FJ980306.1
Plumeria alba PA1
Plumeria alba PA2
Plumeria rubra PR1
Plumeria rubra PR3
Strychnos nux-vomica 111
Strychnos nux-vomica SN122
Strychnos nux-vomica 112
Strychnos potatorum SPT1
Strychnos potatorum 88
Strychnos potatorum 9
Mitragyna parvifolia MPF1
Mitragyna parvifolia MPF3
Mitragyna parvifolia MPF2
Swietenia macrophylla SM1
Mitragyna parvifolia 400
Tabebuia aurea 154
Tabebuia aurea TA2
Tabebuia aurea 54
Tabebuia aurea TA1
Vitex negundo VN1
Vitex negundo VN2
Vitex pinnata FM200126.1
Vitex pinnata VPN1
Hibiscus rosa-sinensis AF460187.1
Azadirachta indica 132
Melia azedarach 140
Melia azedarach FJ518900.1
Ziziphus mauritiana 384
Ziziphus mauritiana JQ627049.1
Aphanamixis polystachya 106
Aphanamixis polystachya AY695584.1
Azadirachta indica 146
Azadirachta indica 46
Swietenia mahogani 199
Blighia sapida 67
Blighia sapida 84
Hibiscus rosa-sinensis 631
Mangifera indica 566
Mangifera indica 631
Pithecellobium dulce 295
Pithecellobium dulce 334
Glycosmis pentaphylla 91
Glycosmis pentaphylla FJ434151.1
Mallotus philippensis 369
Mallotus philippensis DQ866613.1
Sterculia urens SU2
Sterculia urens 39
Swietenia mahogani SMG2
Dalbergia latifolia 361
Dalbergia sissoo DS1
Dalbergia latifolia DL3
Dalbergia latifolia 179
Dalbergia lanceolaria 38
Dalbergia lanceolaria 60
Dalbergia sissoo AF189024.1
Terminalia bellirica FJ381773.1
Swietenia macrophylla SM2
Terminalia muelleri 14
Terminalia muelleri 57
Terminalia muelleri 571
Bauhinia purpurea BP1
Bauhinia purpurea BP2
Bauhinia variegata BV2
Bauhinia variegata BV3
Ficus benghalensis 383
Bauhinia purpurea 76
Ficus racemosa 395
Terminalia bellirica 373
Callistemon citrinus CC1
Strychnos potatorum SPT2
Callistemon lanceolatus CL1
Callistemon citrinus CC3
Callistemon lanceolatus CL3
Callistemon citrinus CC2
Callistemon lanceolatus CL2
Ficus benghalensis HM368185.1
Ficus racemosa HM368195.1
Holoptelea integrifolia 296
Holoptelea integrifolia 312
Holoptelea integrifolia 307
Holoptelea integrifolia 99
Erythrina vespertilio 189
Erythrina vespertilio EV2
Erythrina vespertilio EV1
Erythrina velutina EVL1
Erythrina velutina EVL2
Pongamia pinnata 532
Pongamia pinnata AF467493.1
Butea monosperma 394
Butea monosperma 602
Santalum album 20
Santalum album 372
Caesalpinia mexicana 171
Caesalpinia mexicana CM1
Caesalpinia gilliesii 170
Caesalpinia cacalaco CCA1
Caesalpinia cacalaco CCA2
Caesalpinia gilliesii AY549891.1
Cassia spp 108
Cassia spp 208
Cassia javanica 105
Cassia javanica CN2
Cassia fistula 363
Cassia fistula 308
Cassia fistula CF2
Podocarpus gracilior PDG1
Podocarpus gracilior PDG2

B. ITS set 3

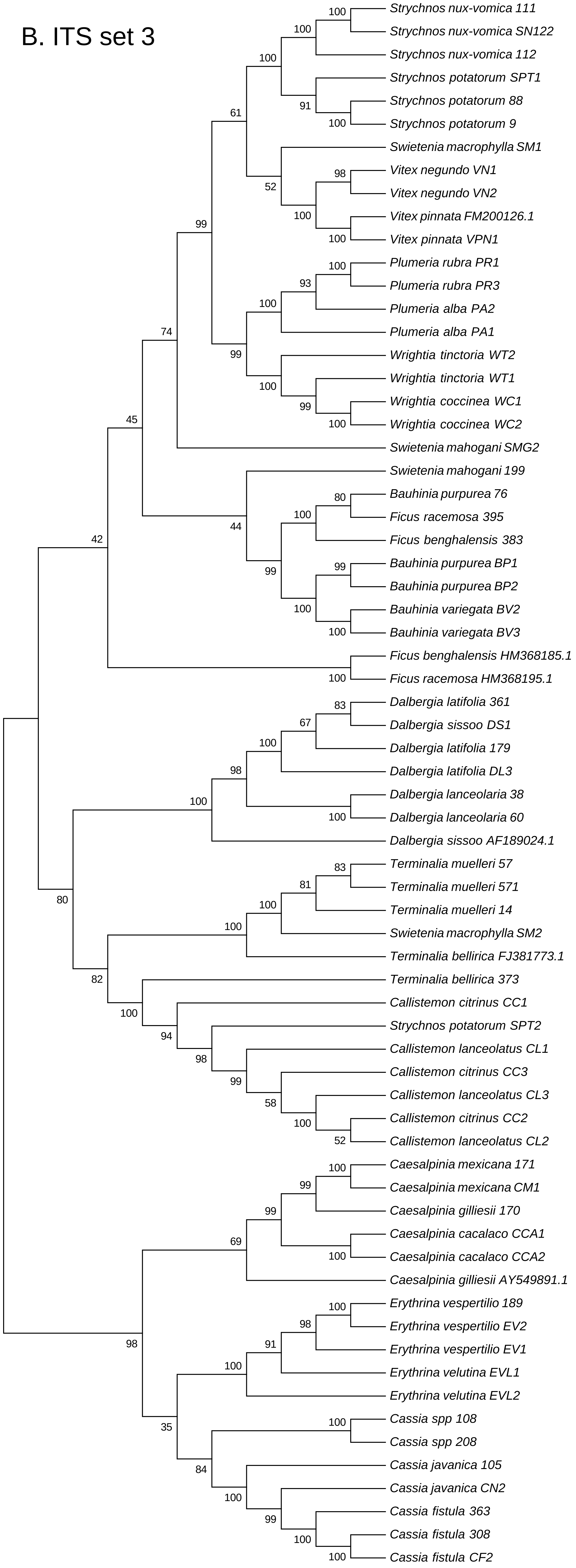
Strychnos nux-vomica 111
Strychnos nux-vomica SN122
Strychnos nux-vomica 112
Strychnos potatorum SPT1
Strychnos potatorum 88
Strychnos potatorum 9
Swietenia macrophylla SM1
Vitex negundo VN1
Vitex negundo VN2
Vitex pinnata FM200126.1
Vitex pinnata VPN1
Plumeria rubra PR1
Plumeria rubra PR3
Plumeria alba PA2
Plumeria alba PA1
Wrightia tinctoria WT2
Wrightia tinctoria WT1
Wrightia coccinea WC1
Wrightia coccinea WC2
Swietenia mahogani SMG2
Swietenia mahogani 199
Bauhinia purpurea 76
Ficus racemosa 395
Ficus benghalensis 383
Bauhinia purpurea BP1
Bauhinia purpurea BP2
Bauhinia variegata BV2
Bauhinia variegata BV3
Ficus benghalensis HM368185.1
Ficus racemosa HM368195.1
Dalbergia latifolia 361
Dalbergia sissoo DS1
Dalbergia latifolia 179
Dalbergia latifolia DL3
Dalbergia lanceolaria 38
Dalbergia lanceolaria 60
Dalbergia sissoo AF189024.1
Terminalia muelleri 57
Terminalia muelleri 571
Terminalia muelleri 14
Swietenia macrophylla SM2
Terminalia bellirica FJ381773.1
Terminalia bellirica 373
Callistemon citrinus CC1
Strychnos potatorum SPT2
Callistemon lanceolatus CL1
Callistemon citrinus CC3
Callistemon lanceolatus CL3
Callistemon citrinus CC2
Callistemon lanceolatus CL2
Caesalpinia mexicana 171
Caesalpinia mexicana CM1
Caesalpinia gilliesii 170
Caesalpinia cacalaco CCA1
Caesalpinia cacalaco CCA2
Caesalpinia gilliesii AY549891.1
Erythrina vespertilio 189
Erythrina vespertilio EV2
Erythrina vespertilio EV1
Erythrina velutina EVL1
Erythrina velutina EVL2
Cassia spp 108
Cassia spp 208
Cassia javanica 105
Cassia javanica CN2
Cassia fistula 363
Cassia fistula 308
Cassia fistula CF2

C. rbcL set 2

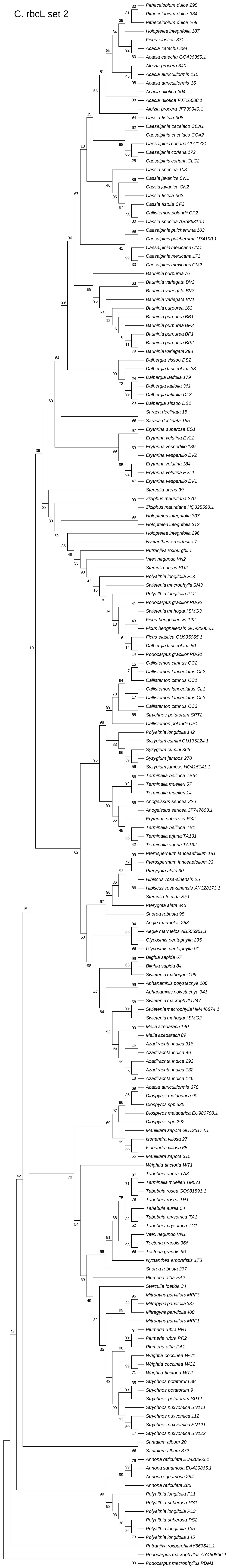
Pithecelobium dulce 295
Pithecelobium dulce 334
Pithecelobium dulce 269
Holoptelea integrifolia 187
Ficus elastica 371
Acacia catechu 294
Acacia catechu GQ436355.1
Albizia procera 340
Acacia auriculiformis 115
Acacia auriculiformis 16
Acacia nilotica 304
Acacia nilotica FJ716688.1
Albizia procera JF739049.1
Cassia fistula 308
Caesalpinia cacalaco CCA1
Caesalpinia cacalaco CCA2
Caesalpinia coriaria CLC1721
Caesalpinia coriaria 172
Caesalpinia coriaria CLC2
Cassia speciea 108
Cassia javanica CN1
Cassia javanica CN2
Cassia fistula 363
Cassia fistula CF2
Callistemon polandii CP2
Cassia speciea AB586310.1
Caesalpinia pulcherrima 103
Caesalpinia pulcherrima U74190.1
Caesalpinia mexicana CM1
Caesalpinia mexicana 171
Caesalpinia mexicana CM2
Bauhinia purpurea 76
Bauhinia variegata BV2
Bauhinia variegata BV3
Bauhinia variegata BV1
Bauhinia purpurea 163
Bauhinia purpurea BB1
Bauhinia purpurea BP3
Bauhinia purpurea BP1
Bauhinia purpurea BP2
Bauhinia variegata 298
Dalbergia sissoo DS2
Dalbergia lanceolaria 38
Dalbergia latifolia 179
Dalbergia latifolia 361
Dalbergia latifolia DL3
Dalbergia sissoo DS1
Saraca declinata 15
Saraca declinata 165
Erythrina suberosa ES1
Erythrina velutina EVL2
Erythrina vespertilio 189
Erythrina vespertilio EV2
Erythrina velutina 184
Erythrina velutina EVL1
Erythrina vespertilio EV1
Sterculia urens 39
Ziziphus mauritiana 270
Ziziphus mauritiana HQ325598.1
Holoptelea integrifolia 307
Holoptelea integrifolia 312
Holoptelea integrifolia 296
Nyctanthes arbortristis 7
Putranjiva roxburghii 1
Vitex negundo VN2
Sterculia urens SU2
Polyalthia longifolia PL4
Swietenia macrophylla SM3
Polyalthia longifolia PL2
Podocarpus gracilior PDG2
Swietenia mahogani SMG3
Ficus benghalensis 122
Ficus benghalensis GU935060.1
Ficus elastica GU935065.1
Dalbergia lanceolaria 60
Podocarpus gracilior PDG1
Callistemon citrinus CC2
Callistemon lanceolatus CL2
Callistemon citrinus CC1
Callistemon lanceolatus CL1
Callistemon lanceolatus CL3
Callistemon citrinus CC3
Strychnos potatorum SPT2
Callistemon polandii CP1
Polyalthia longifolia 142
Syzygium cumini GU135224.1
Syzygium cumini 365
Syzygium jambos 278
Syzygium jambos HQ415141.1
Terminalia bellirica TB64
Terminalia muelleri 57
Terminalia muelleri 14
Anogeissus sericea 226
Anogeissus sericea JF747603.1
Erythrina suberosa ES2
Terminalia bellirica TB1
Terminalia arjuna TA131
Terminalia arjuna TA132
Pterospermum lanceaefolium 181
Pterospermum lanceaefolium 33
Pterygota alata 30
Hibiscus rosa-sinensis 25
Hibiscus rosa-sinensis AY328173.1
Sterculia foetida SF1
Pterygota alata 345
Shorea robusta 95
Aegle marmelos 253
Aegle marmelos AB505961.1
Glycosmis pentaphylla 235
Glycosmis pentaphylla 91
Blighia sapida 67
Blighia sapida 84
Swietenia mahogani 199
Aphanamixis polystachya 106
Aphanamixis polystachya 341
Swietenia macrophylla 247
Swietenia macrophylla HM446874.1
Swietenia mahogani SMG2
Melia azedarach 140
Melia azedarach 89
Azadirachta indica 318
Azadirachta indica 46
Azadirachta indica 293
Azadirachta indica 132
Azadirachta indica 146
Acacia auriculiformis 378
Diospyros malabarica 90
Diospyros spp 335
Diospyros malabarica EU980708.1
Diospyros spp 292
Manilkara zapota GU135174.1
Isonandra villosa 27
Isonandra villosa 65
Manilkara zapota 315
Wrightia tinctoria WT1
Tabebuia aurea TA3
Terminalia muelleri TM571
Tabebuia rosea GQ981891.1
Tabebuia rosea TR1
Tabebuia aurea 54
Tabebuia crysotrica TA1
Tabebuia crysotrica TC1
Vitex negundo VN1
Tectona grandis 366
Tectona grandis 96
Nyctanthes arbortristis 178
Shorea robusta 237
Plumeria alba PA2
Sterculia foetida 34
Mitragyna parvifolia MPF3
Mitragyna parvifolia 337
Mitragyna parvifolia 400
Mitragyna parvifolia MPF1
Plumeria rubra PR1
Plumeria rubra PR2
Plumeria alba PA1
Wrightia coccinea WC1
Wrightia coccinea WC2
Wrightia tinctoria WT2
Strychnos potatorum 88
Strychnos potatorum 9
Strychnos potatorum SPT1
Strychnos nuxvomica SN111
Strychnos nuxvomica 112
Strychnos nuxvomica SN121
Strychnos nuxvomica SN122
Santalum album 20
Santalum album 372
Annona reticulata EU420863.1
Annona squamosa EU420865.1
Annona squamosa 284
Annona reticulata 285
Polyalthia longifolia PL1
Polyalthia suberosa PS1
Polyalthia longifolia PL3
Polyalthia suberosa PS2
Polyalthia longifolia 135
Polyalthia longifolia 145
Putranjiva roxburghii AY663641.1
Podocarpus macrophyllus AY450866.1
Podocarpus macrophyllus PDM1

D. rbcL set 3

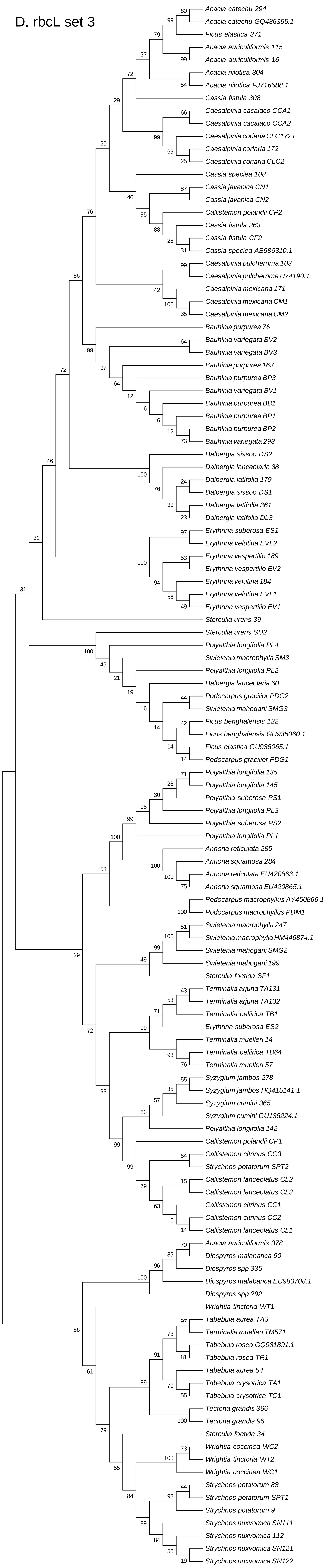
Acacia catechu 294
Acacia catechu GQ436355.1
Ficus elastica 371
Acacia auriculiformis 115
Acacia auriculiformis 16
Acacia nilotica 304
Acacia nilotica FJ716688.1
Cassia fistula 308
Caesalpinia cacalaco CCA1
Caesalpinia cacalaco CCA2
Caesalpinia coriaria CLC1721
Caesalpinia coriaria 172
Caesalpinia coriaria CLC2
Cassia speciea 108
Cassia javanica CN1
Cassia javanica CN2
Callistemon polandii CP2
Cassia fistula 363
Cassia fistula CF2
Cassia speciea AB586310.1
Caesalpinia pulcherrima 103
Caesalpinia pulcherrima U74190.1
Caesalpinia mexicana 171
Caesalpinia mexicana CM1
Caesalpinia mexicana CM2
Bauhinia purpurea 76
Bauhinia variegata BV2
Bauhinia variegata BV3
Bauhinia purpurea 163
Bauhinia purpurea BP3
Bauhinia variegata BV1
Bauhinia purpurea BB1
Bauhinia purpurea BP1
Bauhinia purpurea BP2
Bauhinia variegata 298
Dalbergia sissoo DS2
Dalbergia lanceolaria 38
Dalbergia latifolia 179
Dalbergia sissoo DS1
Dalbergia latifolia 361
Dalbergia latifolia DL3
Erythrina suberosa ES1
Erythrina velutina EVL2
Erythrina vespertilio 189
Erythrina vespertilio EV2
Erythrina velutina 184
Erythrina velutina EVL1
Erythrina vespertilio EV1
Sterculia urens 39
Sterculia urens SU2
Polyalthia longifolia PL4
Swietenia macrophylla SM3
Polyalthia longifolia PL2
Dalbergia lanceolaria 60
Podocarpus gracilior PDG2
Swietenia mahogani SMG3
Ficus benghalensis 122
Ficus benghalensis GU935060.1
Ficus elastica GU935065.1
Podocarpus gracilior PDG1
Polyalthia longifolia 135
Polyalthia longifolia 145
Polyalthia suberosa PS1
Polyalthia longifolia PL3
Polyalthia suberosa PS2
Polyalthia longifolia PL1
Annona reticulata 285
Annona squamosa 284
Annona reticulata EU420863.1
Annona squamosa EU420865.1
Podocarpus macrophyllus AY450866.1
Podocarpus macrophyllus PDM1
Swietenia macrophylla 247
Swietenia macrophylla HM446874.1
Swietenia mahogani SMG2
Swietenia mahogani 199
Sterculia foetida SF1
Terminalia arjuna TA131
Terminalia arjuna TA132
Terminalia bellirica TB1
Erythrina suberosa ES2
Terminalia muelleri 14
Terminalia bellirica TB64
Terminalia muelleri 57
Syzygium jambos 278
Syzygium jambos HQ415141.1
Syzygium cumini 365
Syzygium cumini GU135224.1
Polyalthia longifolia 142
Callistemon polandii CP1
Callistemon citrinus CC3
Strychnos potatorum SPT2
Callistemon lanceolatus CL2
Callistemon lanceolatus CL3
Callistemon citrinus CC1
Callistemon citrinus CC2
Callistemon lanceolatus CL1
Acacia auriculiformis 378
Diospyros malabarica 90
Diospyros spp 335
Diospyros malabarica EU980708.1
Diospyros spp 292
Wrightia tinctoria WT1
Tabebuia aurea TA3
Terminalia muelleri TM571
Tabebuia rosea GQ981891.1
Tabebuia rosea TR1
Tabebuia aurea 54
Tabebuia crysotrica TA1
Tabebuia crysotrica TC1
Tectona grandis 366
Tectona grandis 96
Sterculia foetida 34
Wrightia coccinea WC2
Wrightia tinctoria WT2
Wrightia coccinea WC1
Strychnos potatorum 88
Strychnos potatorum SPT1
Strychnos potatorum 9
Strychnos nuxvomica SN111
Strychnos nuxvomica 112
Strychnos nuxvomica SN121
Strychnos nuxvomica SN122

E. trnH-psbA set2

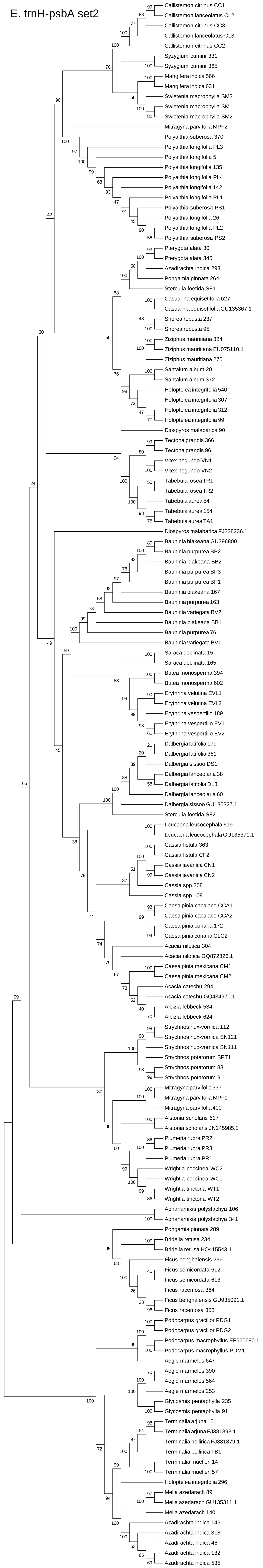

F. trnH-psbA set 3

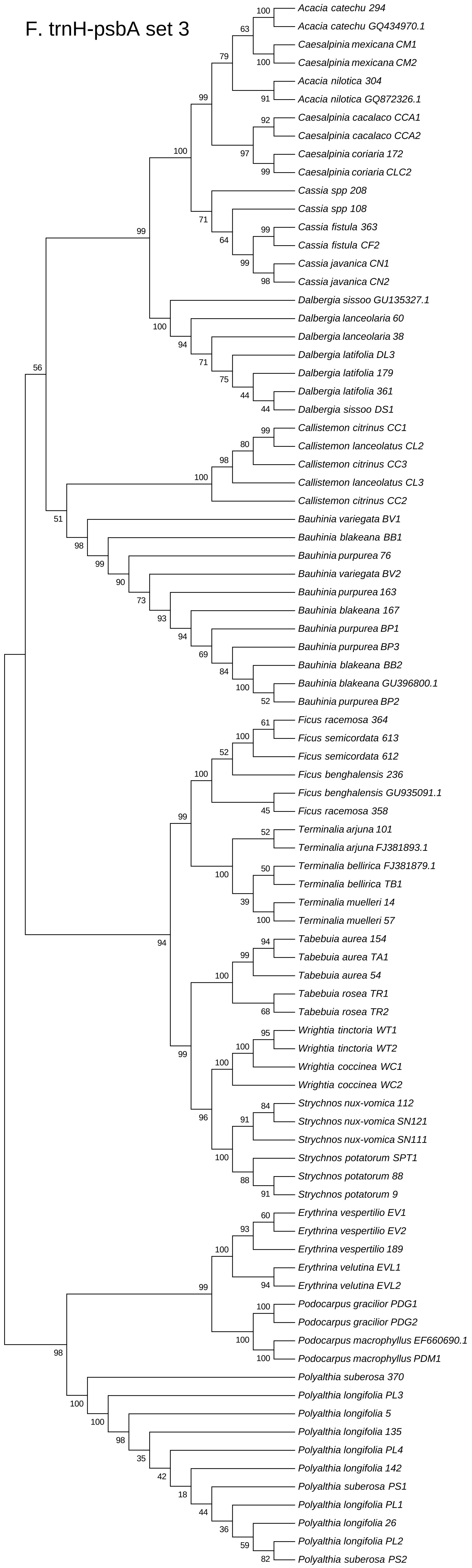

G. ITS-2 set2

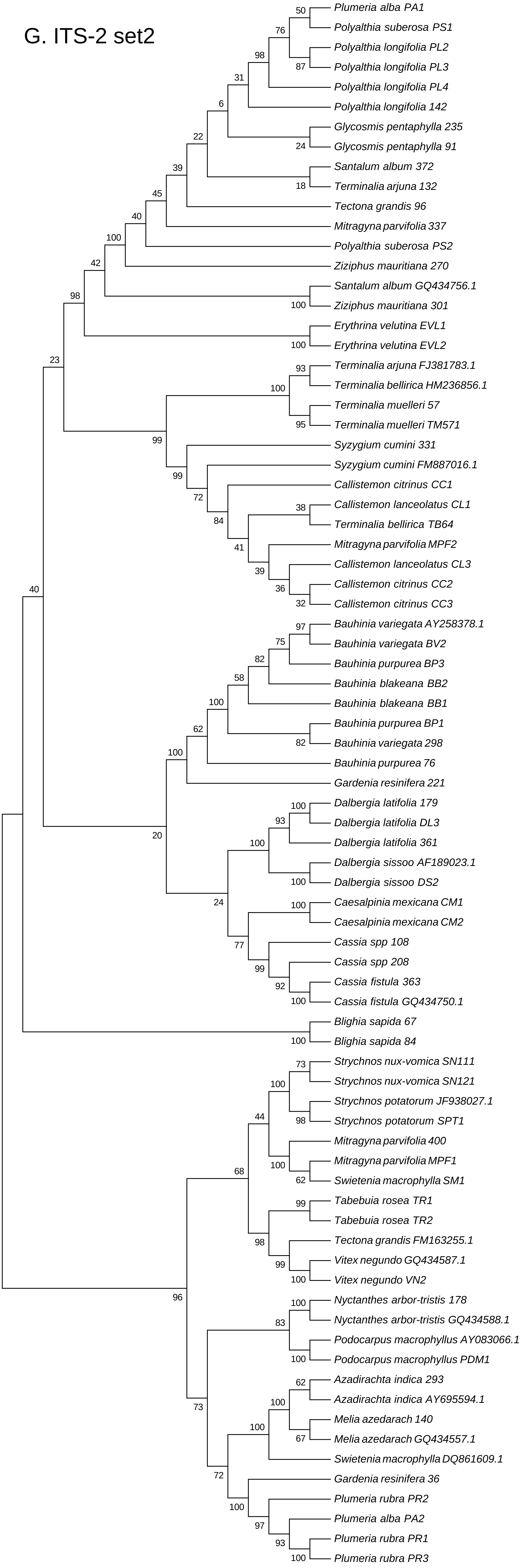
Plumeria alba PA1
Polyalthia suberosa PS1
Polyalthia longifolia PL2
Polyalthia longifolia PL3
Polyalthia longifolia PL4
Polyalthia longifolia 142
Glycosmis pentaphylla 235
Glycosmis pentaphylla 91
Santalum album 372
Terminalia arjuna 132
Tectona grandis 96
Mitragyna parvifolia 337
Polyalthia suberosa PS2
Ziziphus mauritiana 270
Santalum album GQ434756.1
Ziziphus mauritiana 301
Erythrina velutina EVL1
Erythrina velutina EVL2
Terminalia arjuna FJ381783.1
Terminalia bellirica HM236856.1
Terminalia muelleri 57
Terminalia muelleri TM571
Syzygium cumini 331
Syzygium cumini FM887016.1
Callistemon citrinus CC1
Callistemon lanceolatus CL1
Terminalia bellirica TB64
Mitragyna parvifolia MPF2
Callistemon lanceolatus CL3
Callistemon citrinus CC2
Callistemon citrinus CC3
Bauhinia variegata AY258378.1
Bauhinia variegata BV2
Bauhinia purpurea BP3
Bauhinia blakeana BB2
Bauhinia blakeana BB1
Bauhinia purpurea BP1
Bauhinia variegata 298
Bauhinia purpurea 76
Gardenia resinifera 221
Dalbergia latifolia 179
Dalbergia latifolia DL3
Dalbergia latifolia 361
Dalbergia sissoo AF189023.1
Dalbergia sissoo DS2
Caesalpinia mexicana CM1
Caesalpinia mexicana CM2
Cassia spp 108
Cassia spp 208
Cassia fistula 363
Cassia fistula GQ434750.1
Blighia sapida 67
Blighia sapida 84
Strychnos nux-vomica SN111
Strychnos nux-vomica SN121
Strychnos potatorum JF938027.1
Strychnos potatorum SPT1
Mitragyna parvifolia 400
Mitragyna parvifolia MPF1
Swietenia macrophylla SM1
Tabebuia rosea TR1
Tabebuia rosea TR2
Tectona grandis FM163255.1
Vitex negundo GQ434587.1
Vitex negundo VN2
Nyctanthes arbor-tristis 178
Nyctanthes arbor-tristis GQ434588.1
Podocarpus macrophyllus AY083066.1
Podocarpus macrophyllus PDM1
Azadirachta indica 293
Azadirachta indica AY695594.1
Melia azedarach 140
Melia azedarach GQ434557.1
Swietenia macrophylla DQ861609.1
Gardenia resinifera 36
Plumeria rubra PR2
Plumeria alba PA2
Plumeria rubra PR1
Plumeria rubra PR3

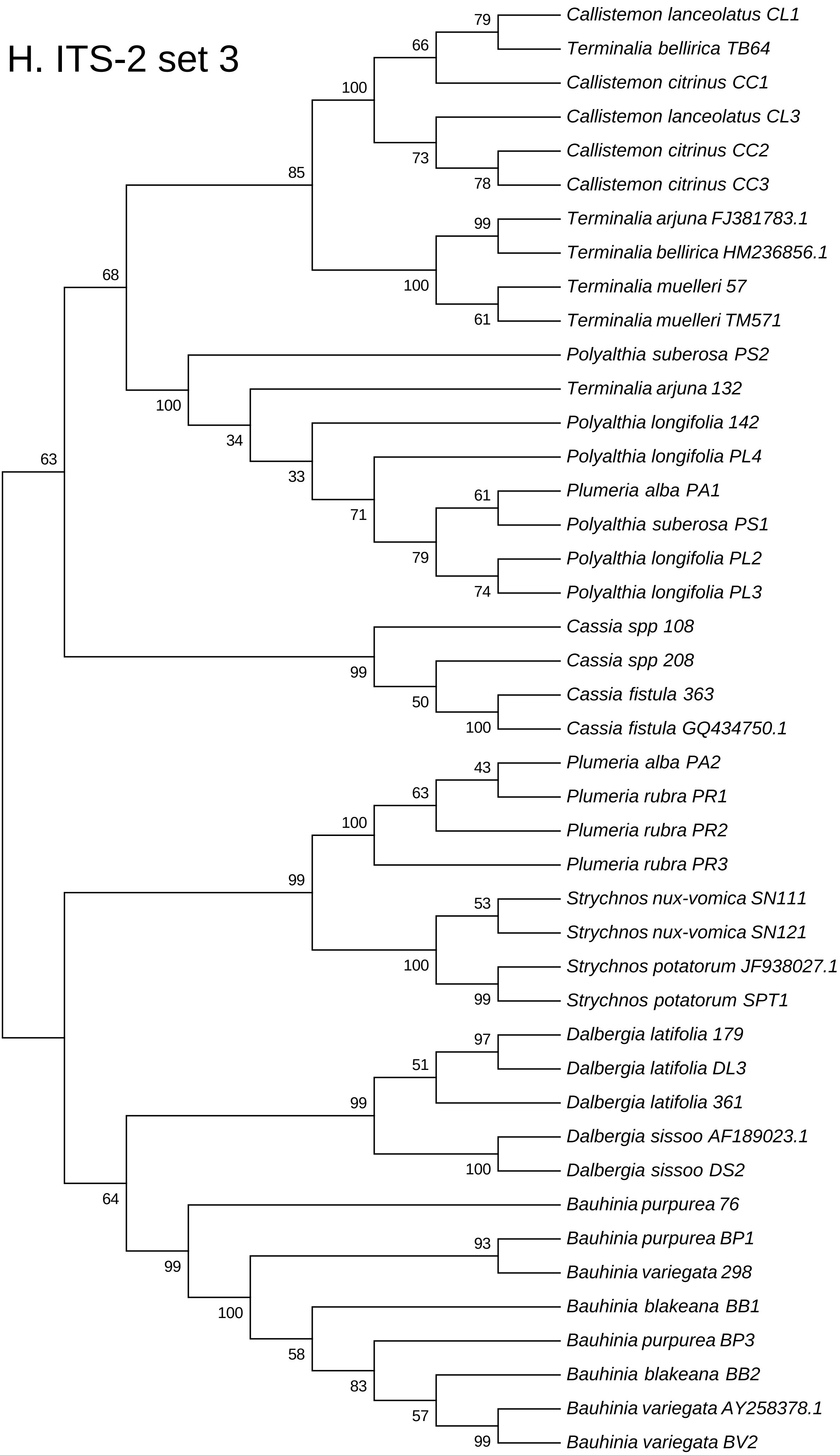
H. ITS-2 set 3
Callistemon lanceolatus CL1
Terminalia bellirica TB64
Callistemon citrinus CC1
Callistemon lanceolatus CL3
Callistemon citrinus CC2
Callistemon citrinus CC3
Terminalia arjuna FJ381783.1
Terminalia bellirica HM236856.1
Terminalia muelleri 57
Terminalia muelleri TM571
Polyalthia suberosa PS2
Terminalia arjuna 132
Polyalthia longifolia 142
Polyalthia longifolia PL4
Plumeria alba PA1
Polyalthia suberosa PS1
Polyalthia longifolia PL2
Polyalthia longifolia PL3
Cassia spp 108
Cassia spp 208
Cassia fistula 363
Cassia fistula GQ434750.1
Plumeria alba PA2
Plumeria rubra PR1
Plumeria rubra PR2
Plumeria rubra PR3
Strychnos nux-vomica SN111
Strychnos nux-vomica SN121
Strychnos potatorum JF938027.1
Strychnos potatorum SPT1
Dalbergia latifolia 179
Dalbergia latifolia DL3
Dalbergia latifolia 361
Dalbergia sissoo AF189023.1
Dalbergia sissoo DS2
Bauhinia purpurea 76
Bauhinia purpurea BP1
Bauhinia variegata 298
Bauhinia blakeana BB1
Bauhinia purpurea BP3
Bauhinia blakeana BB2
Bauhinia variegata AY258378.1
Bauhinia variegata BV2

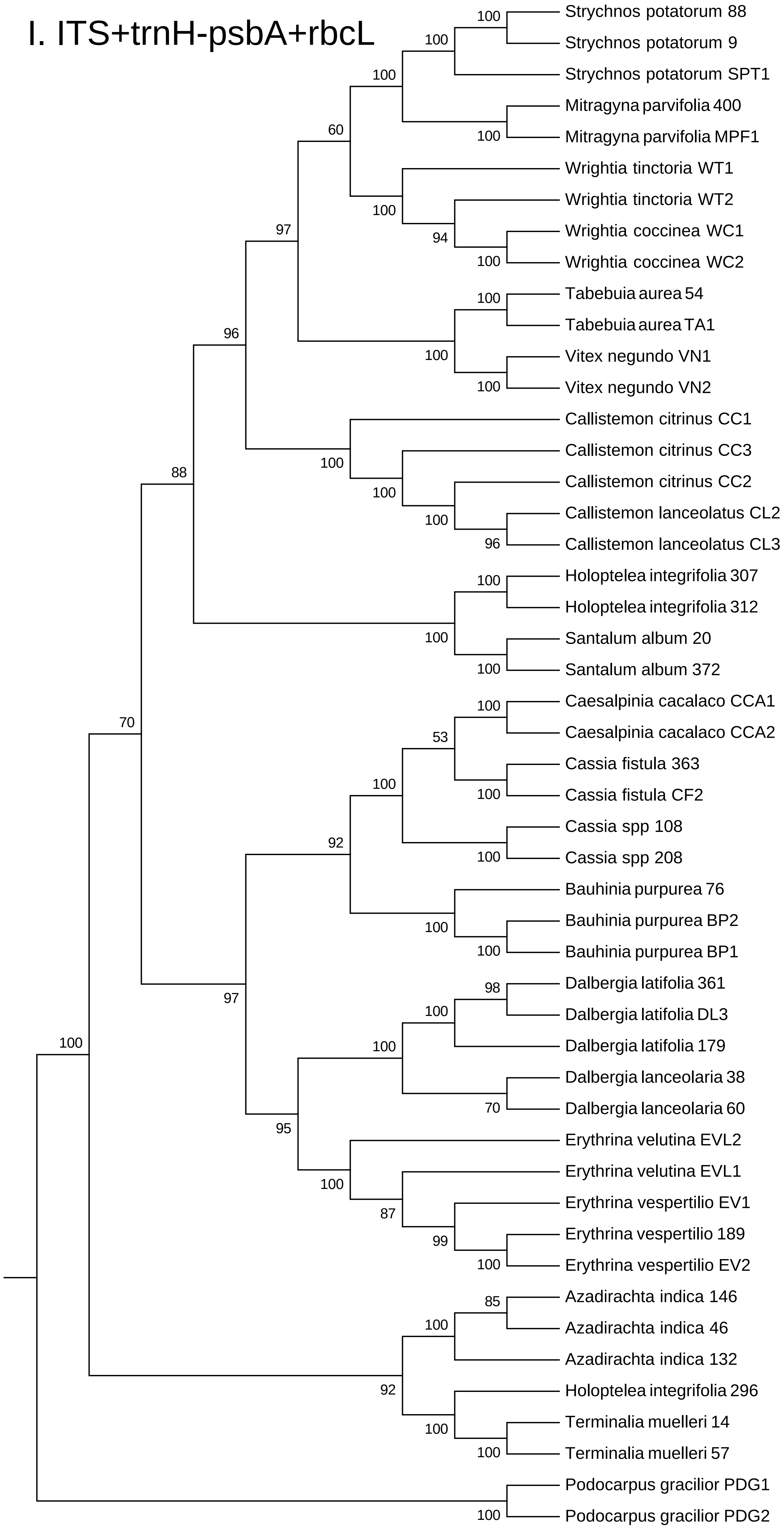
I. ITS+trnH-psbA+rbcL
Strychnos potatorum 88
Strychnos potatorum 9
Strychnos potatorum SPT1
Mitragyna parvifolia 400
Mitragyna parvifolia MPF1
Wrightia tinctoria WT1
Wrightia tinctoria WT2
Wrightia coccinea WC1
Wrightia coccinea WC2
Tabebuia aurea 54
Tabebuia aurea TA1
Vitex negundo VN1
Vitex negundo VN2
Callistemon citrinus CC1
Callistemon citrinus CC3
Callistemon citrinus CC2
Callistemon lanceolatus CL2
Callistemon lanceolatus CL3
Holoptelea integrifolia 307
Holoptelea integrifolia 312
Santalum album 20
Santalum album 372
Caesalpinia cacalaco CCA1
Caesalpinia cacalaco CCA2
Cassia fistula 363
Cassia fistula CF2
Cassia spp 108
Cassia spp 208
Bauhinia purpurea 76
Bauhinia purpurea BP2
Bauhinia purpurea BP1
Dalbergia latifolia 361
Dalbergia latifolia DL3
Dalbergia latifolia 179
Dalbergia lanceolaria 38
Dalbergia lanceolaria 60
Erythrina velutina EVL2
Erythrina velutina EVL1
Erythrina vespertilio EV1
Erythrina vespertilio 189
Erythrina vespertilio EV2
Azadirachta indica 146
Azadirachta indica 46
Azadirachta indica 132
Holoptelea integrifolia 296
Terminalia muelleri 14
Terminalia muelleri 57
Podocarpus gracilior PDG1
Podocarpus gracilior PDG2
