# Supplementary material for: The Internal Transcribed Spacer (ITS) Region and trnhH-psbA Are Suitable Candidate Loci for DNA Barcoding of Tropical Tree Species of India
Source: PLoS One. 2013 Feb 27;8(2):e57934. doi: 10.1371/journal.pone.0057934 (PMC3584017; doi:10.1371/journal.pone.0057934)
Supplement: Table S2 — List of primer sequences (5′-3′) and their references used in this study. (PDF) [file pone.0057934.s003.pdf]

Table S2: List of primer sequences (5′-3′) and their references used in this study

Locus	Direction	Sequence	Reference
ITS5a	Forward	CCTTATCATTTAGAGGAAGGA	(22)
ITS4	Reverse	TCCTCCGCTTATTGATATGC	(22)
ITS2	Forward	ATGCGATACTTGGTGTGAAT	(45)
	Reverse	GACGCTTCTCCAGACTACAAT	
matK 2.1a	Forward	ATCCATCTGGAAATCTTAGTTC	http://www.kew.org/barcoding/protocols.html
matK-NBRI	Forward	TCCCCATCCATCTGGAA	(35)
*matK3.*2r	Reverse	ATCTATCGATAATATCAGAAT	http://www.kew.org/barcoding/protocols.html
*rbcL*1F	Forward	ATGTCACCACAAACAGAAAC	(22)
*rbcL*724r	Reverse	TCGCATGTACCTGCAGTAGC	(22)
trnH-psbA	*trnH*	CGCGCATGGTGGATTCACAATCC	(22)
	psbA	GTTATGCATGAACGTAATGCT	(22)
