# Supplementary material for: The Internal Transcribed Spacer (ITS) Region and trnhH-psbA Are Suitable Candidate Loci for DNA Barcoding of Tropical Tree Species of India
Source: PLoS One. 2013 Feb 27;8(2):e57934. doi: 10.1371/journal.pone.0057934 (PMC3584017; doi:10.1371/journal.pone.0057934)
Supplement: Table S3 — Kruskal-Wallis test with Dunn's multiple comparison to compare inter (A) and intraspecific (B) variability for each individual locus. (DOCX) [file pone.0057934.s004.docx]

**Table S3: Kruskal-Wallis test with Dunn's multiple comparison to compare inter (A) and intraspecific (B) variability for each individual locus**

A

| **Kruskal-Wallis test between Interspecific distances (set 2)** |  |  |  |  |
| --- | --- | --- | --- | --- |
| P value | < 0.0001 |  |  |  |
| Exact or approximate P value? | Gaussian Approximation |  |  |  |
| P value summary | *** |  |  |  |
| Do the medians vary signif. (P < 0.05) | Yes |  |  |  |
| Number of groups | 4 |  |  |  |
| Kruskal-Wallis statistic | 182.5 |  |  |  |
|  |  |  |  |  |
| Dunn's Multiple Comparison Test | Difference in rank sum | Significant? P < 0.05? | Summary | Result |
| ITS vs rbcL | 75.79 | Yes | *** | ITS >> rbcL |
| ITS vs trnH-psbA | -69.71 | Yes | *** | ITS << trnH-psbA |
| ITS vs ITS-2 | -14.14 | No | ns | ITS = ITS-2 |
| rbcL vs trnH-psbA | -145.5 | Yes | *** | rbcL << trnH-psbA |
| rbcL vs ITS-2 | -89.93 | Yes | *** | rbcL << ITS-2 |
| trnH-psbA vs ITS-2 | 55.57 | Yes | *** | trnH-psbA >> ITS-2 |

B

| **Kruskal-Wallis test between Intraspecific distances (set 2)** |  |  |  |  |
| --- | --- | --- | --- | --- |
| P value | 0.0004 |  |  |  |
| Exact or approximate P value? | Gaussian Approximation |  |  |  |
| P value summary | *** |  |  |  |
| Do the medians vary signif. (P < 0.05) | Yes |  |  |  |
| Number of groups | 4 |  |  |  |
| Kruskal-Wallis statistic | 18.11 |  |  |  |
|  |  |  |  |  |
| Dunn's Multiple Comparison Test | Difference in rank sum | Significant? P < 0.05? | Summary |  |
| ITS vs rbcL | 22.74 | No | ns | ITS = rbcL |
| ITS vs trnH-psbA | 26.65 | No | ns | ITS = trnH-psbA |
| ITS vs ITS-2 | -22.46 | No | ns | ITS = ITS-2 |
| rbcL vs trnH-psbA | 3.914 | No | ns | rbcL = trnH-psbA |
| rbcL vs ITS-2 | -45.19 | Yes | ** | rbcL << ITS-2 |
| trnH-psbA vs ITS-2 | -49.11 | Yes | ** | trnH-psbA << ITS-2 |

C

| **Kruskal-Wallis test between Interspecific distances (set 3)** |  |  |  |  |
| --- | --- | --- | --- | --- |
| P value | < 0.0001 |  |  |  |
| Exact or approximate P value? | Gaussian Approximation |  |  |  |
| P value summary | *** |  |  |  |
| Do the medians vary signif. (P < 0.05) | Yes |  |  |  |
| Number of groups | 4 |  |  |  |
| Kruskal-Wallis statistic | 109.8 |  |  |  |
|  |  |  |  |  |
| Dunn's Multiple Comparison Test | Difference in rank sum | Significant? P < 0.05? | Summary | Result |
| ITS vs rbcL | 49.33 | Yes | *** | ITS >> rbcL |
| ITS vs trnH-psbA | -37.05 | Yes | *** | ITS << trnH-psbA |
| ITS vs ITS-2 | 1.716 | No | ns | ITS = ITS-2 |
| rbcL vs trnH-psbA | -86.38 | Yes | *** | rbcL << trnH-psbA |
| rbcL vs ITS-2 | -47.61 | Yes | *** | rbcL << ITS-2 |
| trnH-psbA vs ITS-2 | 38.77 | Yes | ** | trnH-psbA >> ITS-2 |

D

| **Kruskal-Wallis test between Intraspecific distances (set 3)** |  |  |  |  |
| --- | --- | --- | --- | --- |
| P value | 0.0119 |  |  |  |
| Exact or approximate P value? | Gaussian Approximation |  |  |  |
| P value summary | * |  |  |  |
| Do the medians vary signif. (P < 0.05) | Yes |  |  |  |
| Number of groups | 4 |  |  |  |
| Kruskal-Wallis statistic | 10.98 |  |  |  |
|  |  |  |  |  |
| Dunn's Multiple Comparison Test | Difference in rank sum | Significant? P < 0.05? | Summary | Result |
| ITS vs rbcL | 16.84 | No | ns | ITS = rbcL |
| ITS vs trnH-psbA | 22.41 | No | ns | ITS = trnH-psbA |
| ITS vs ITS-2 | -6.547 | No | ns | ITS = ITS-2 |
| rbcL vs trnH-psbA | 5.564 | No | ns | rbcL = trnH-psbA |
| rbcL vs ITS-2 | -23.39 | No | ns | rbcL = ITS-2 |
| trnH-psbA vs ITS-2 | -28.95 | Yes | * | trnH-psbA << ITS-2 |
