# Supplementary material for: The Internal Transcribed Spacer (ITS) Region and trnhH-psbA Are Suitable Candidate Loci for DNA Barcoding of Tropical Tree Species of India
Source: PLoS One. 2013 Feb 27;8(2):e57934. doi: 10.1371/journal.pone.0057934 (PMC3584017; doi:10.1371/journal.pone.0057934)
Supplement: Table S4 — Wilcoxon matched-pair test to compare between minimum inter and maximum intraspecific p-distance differences of different loci. Set 2, (A); Set 3, (B). (DOCX) [file pone.0057934.s005.docx]

**Table S4: Wilcoxon matched-pair test to compare between minimum interspecific and maximum intraspecific p-distance differences of different loci and their combinations.**

**A**

| Set 2 | P value | P value summary | Sum of positive, negative ranks | Sum of signed ranks (W) | Result |
| --- | --- | --- | --- | --- | --- |
|  |  |  |  |  |  |
| ITS | 0.6779 | ns | 629.0 , -547.0 | 82 | Interspecific p-distance = Intraspecific p-diatance |
| *rbcL* | 0.0072 | sd | 732.5 , -1614 | -881 | Interspecific p-distance << Intraspecific p-diatance |
| *trnH-psbA* | 0.0071 | sd | 1281 , -549.0 | 732 | Interspecific p-distance >> Intraspecific p-diatance |
| ITS-2 | 0.4967 | ns | 273.0 , -357.0 | -84 | Interspecific p-distance = Intraspecific p-diatance |
| ITS+*trnH-psbA* | 0.0007 | sd | 214.0 , -17.00 | 197 | Interspecific p-distance >> Intraspecific p-diatance |
| ITS+*trnH-psbA+rbcL* | 0.0056 | sd | 266.0 , -59.00 | 229 | Interspecific p-distance >> Intraspecific p-diatance |
|  |  |  |  |  |  |
| **B** |  |  |  |  |  |
|  |  |  |  |  |  |
| Set 3 | P value | P value summary | Sum of positive, negative ranks | Sum of signed ranks (W) | Result |
|  |  |  |  |  |  |
| ITS | 0.3305 | ns | 172.0 , -263.0 | -91 | Interspecific p-distance = Intraspecific p-diatance |
| *rbcL* | 0.0009 | sd | 186.5 , -716.5 | -530 | Interspecific p-distance << Intraspecific p-diatance |
| *trnH-psbA* | 0.1559 | ns | 381.0 , -214.0 | 167 | Interspecific p-distance = Intraspecific p-diatance |
| ITS-2 | 0.6013 | ns | 73.00 , -98.00 | -25 | Interspecific p-distance = Intraspecific p-diatance |
