# Supplementary material for: The Internal Transcribed Spacer (ITS) Region and trnhH-psbA Are Suitable Candidate Loci for DNA Barcoding of Tropical Tree Species of India
Source: PLoS One. 2013 Feb 27;8(2):e57934. doi: 10.1371/journal.pone.0057934 (PMC3584017; doi:10.1371/journal.pone.0057934)
Supplement: Table S5 — The status of ITS and trnH-psbA sequences with respect to studied tree species in NCBI nucleotide database as on 03.01.2013. The table shows the species name whose ITS and/or trnH-psbA sequences are abscent in the database. (DOCX) [file pone.0057934.s006.docx]

**Table S5: The status of ITS and *trnH-psbA* sequences with respect to studied tree species in NCBI nucleotide database as on 03.01.2013.** The table shows the species name whose ITS and/or *trnH-psbA* sequences are abscent in the database.

| ITS | *trnH-psbA* |
| --- | --- |
| *Acacia auriculiformis* | *Acacia lenticularis* |
| *Albizia lucida* | *Acacia nilotica* |
| *Albizia lucidior* | *Acacia spp* |
| *Alstonia macrophylla* | *Aegle marmelos* |
| *Anthocephalus cadamba* | *Albizia amara* |
| *Aphanamixis polystachya* | *Albizia lucida* |
| *Butea monosperma* | *Albizia lucidior* |
| *Caesalpinia cacalaco* | *Annona reticulata* |
| *Caesalpinia coriaria* | *Aphanamixis polystachya* |
| *Caesalpinia gilliesii* | *Azadirachta indica* |
| *Caesalpinia pulcherrima* | *Bauhinia purpurea* |
| *Caesalpinia spp* | *Bauhinia variabilis* |
| *Callistemon citrinus* | *Blighia sapida* |
| *Cassia sp.* | *Butea monosperma* |
| *Dalbergia lanceolaria* | *Caesalpinia cacalaco* |
| *Dalbergia latifolia* | *Caesalpinia coriaria* |
| *Derris ovalifolia* | *Caesalpinia mexicana* |
| *Desmodium oojeinense* | *Callistemon citrinus* |
| *Ehretia laevis* | *Callistemon lanceolatus* |
| *Erythrina lithosperma* | *Callistemon polandii* |
| *Erythrina suberosa* | *Cassia nodosa* |
| *Erythrina subumbrans* | *Cassia spp* |
| *Erythrina velutina* | *Dalbergia lanceolaria* |
| *Erythrina vespertilio* | *Dalbergia latifolia* |
| *Ficus infectoria* | *Derris ovalifolia* |
| *Handroanthus impetiginosus* | *Desmodium oojeinense* |
| *Holoptelea integrifolia* | *Diospyros spp* |
| *Ixora pavetta* | *Erythrina suberosa* |
| *Lagerstroemia hirsuta* | *Erythrina subumbrans* |
| *Lagerstroemia parviflora* | *Erythrina velutina* |
| *Lagerstroemia reginae* | *Erythrina vespertilio* |
| *Manilkara hexandra* | *Eucalyptus tereticornis* |
| *Manilkara zapota* | *Handroanthus impetiginosus* |
| *Millettia ovalifolia* | *Holarrhena antidysentrica* |
| *Millettia peguensis* | *Holoptelea integrifolia* |
| *Mitragyna parvifolia* | *Jacaranda cuspidifolia* |
| *Podocarpus gracilior* | *Jacaranda mimosifolia* |
| *Polyalthia longifolia* | *Lagerstroemia flos-reginae* |
| *Polyalthia suberosa* | *Lagerstroemia hirsuta* |
| *Pterospermum acerifolium* | *Mitragyna parvifolia* |
| *Pterygota alata* | *Pithecellobium dulce* |
| *Sterculia urens* | *Plumeria rubra* |
| *Strychnos potatorum* | *Podocarpus gracilior* |
| *Swietenia mahogani* | *Polyalthia longifolia* |
| *Tabebuia argentea* | *Polyalthia suberosa* |
| *Tabebuia aurea* | *Pongamia pinnata* |
| *Tabebuia chrysotricha* | *Pterospermum acerifolium* |
| *Tabebuia palmeri* | *Pterygota alata* |
| *Tabebuia rosea* | *Saraca declinata* |
| *Tabernaemontana elegans* | *Shorea robusta* |
| *Terminalia catappa* | *Sterculia foetida* |
| *Vitex negundo* | *Strychnos potatorum* |
| *Wrightia coccinea* | *Swietenia mahogani* |
| *Wrightia tinctoria* | *Tabebuia argentea* |
|  | *Tabebuia aurea* |
|  | *Tabebuia palmeri* |
|  | *Tabebuia rosea* |
|  | *Tectona grandis* |
|  | *Terminalia bellirica* |
|  | *Vitex negundo* |
|  | *Vitex pinnata* |
|  | *Wrightia coccinea* |
|  | *Wrightia tinctoria* |
|  | *Ziziphus nummularia* |

|  |  |
| --- | --- |
|  |  |
